# Manganese-Catalyzed Hydrogenation of Ketones under Mild and Base-free Conditions

**DOI:** 10.1021/acs.organomet.1c00161

**Published:** 2021-04-22

**Authors:** Stefan Weber, Julian Brünig, Luis F. Veiros, Karl Kirchner

**Affiliations:** † Institute of Applied Synthetic Chemistry, Vienna University of Technology, Getreidemarkt 9, Vienna A-1060, Austria; ‡ Centro de Química Estrutural and Departamento de Engenharia Química, Instituto Superior Técnico, Universidade de Lisboa, Av Rovisco Pais, Lisboa 1049-001, Portugal

## Abstract

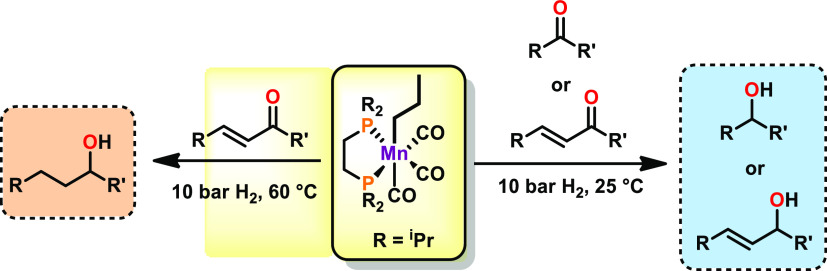

In this paper, several Mn(I) complexes were applied as catalysts for the homogeneous hydrogenation of ketones. The most active precatalyst is the bench-stable alkyl bisphosphine Mn(I) complex *fac*-[Mn(dippe) (CO)_3_(CH_2_CH_2_CH_3_)]. The reaction proceeds at room temperature under base-free conditions with a catalyst loading of 3 mol % and a hydrogen pressure of 10 bar. A temperature-dependent selectivity for the reduction of α,β-unsaturated carbonyls was observed. At room temperature, the carbonyl group was selectively hydrogenated, while the C=C bond stayed intact. At 60 °C, fully saturated systems were obtained. A plausible mechanism based on DFT calculations which involves an inner-sphere hydride transfer is proposed.

## Introduction

The catalytic reduction of polar multiple bonds *via* molecular hydrogen plays a significant role in modern synthetic organic chemistry. Within this context, the use of catalytic procedures in combination with hydrogen gas displays an attractive option to develop efficient and cleaner processes.^1^ In the last few years, well-defined Mn(I) complexes were introduced as powerful players in the field of sustainable hydrogenation chemistry,^2^ being active for the hydrogenation of not only aldehydes,^3^ ketones,^4^ esters,^5^ CO_2_,^6^ and carbonates^7^ but also nitrogen-containing compounds such as imines,^8^ nitriles,^9^ amides,^10^ and heterocycles.^11^

**Scheme 1:**
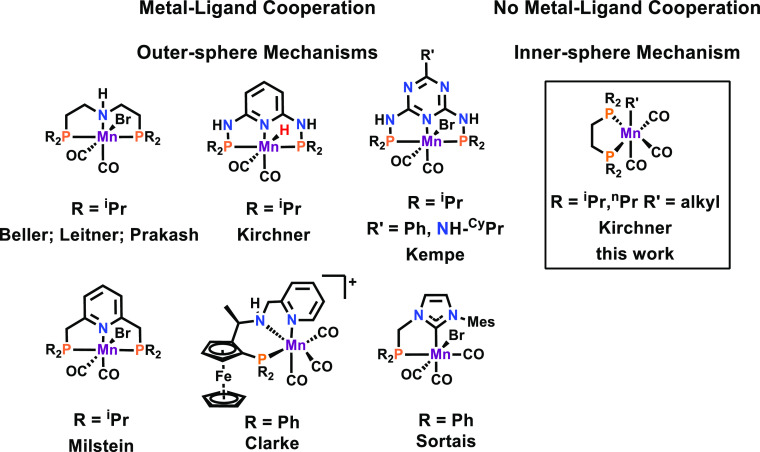
Selected Mn(I) Precatalysts for Hydrogenation Reactions

It is interesting to note that many of these transition-metal-catalyzed hydrogenations rely on metal–ligand bifunctional catalysis (metal–ligand cooperation), where complexes contain electronically coupled hydride and acidic hydrogen atoms. An effective way of bond activation by metal–ligand cooperation involves aromatization/dearomatization of the ligand in pincer complexes in which a central pyridine-based backbone is connected with −CH_2_PR_2_ and/or −CH_2_NR_2_ substituents. This has resulted in the development of novel and unprecedented iron and manganese catalysis, where this type of cooperation plays a key role in the heterolytic cleavage of H_2_. An overview of well-defined manganese complexes for hydrogenation reactions is depicted in Scheme 1.

**Scheme 2:**
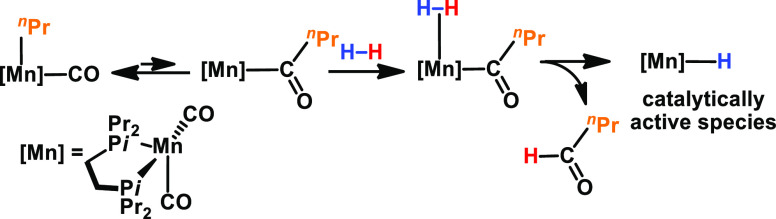
Formation of the Catalytically Active Species Upon Reaction With Dihydrogen

An alternative way to activate dihydrogen was recently described by our group. We took advantage of the fact that Mn(I) alkyl carbonyl complexes are known to undergo insertions to form highly reactive acyl intermediates (a well-known reaction in organometallic chemistry^12^) which are able to activate dihydrogen, thereby forming the 16e^–^ Mn(I) hydride catalysts (Scheme 2). Accordingly, bisphosphine manganese tricarbonyl complexes containing alkyl ligands could be employed for the additive-free hydrogenation of alkenes and nitriles.^13,9c^

Here, we describe an additive-free hydrogenation of ketones at room temperature, utilizing Mn(I) alkyl carbonyl complexes *fac*-[Mn(dpre) (CO)_3_(CH_3_)] (dpre = 1,2-*bis*(di-*n*-propylphosphino)ethane, *fac*-[Mn(dpre) (CO)_3_(CH_2_CH_2_CH_3_)] (**2**) and *fac*-[Mn(dippe) (CO)_3_(CH_2_CH_2_CH_3_) (dippe = 1,2-*bis*(di-*iso*-propylphosphino)ethane) (**3**).

## Results and Discussion

The catalytic performance of manganese(I) alkyl complexes **1–3** for the hydrogenation of ketones was evaluated. The experiments were performed using Et_2_O as the solvent at 25 °C and 50 bar H_2_ pressure and 4-fluoroacetophenone as the model substrate to find the most active catalyst and optimal hydrogenation reaction conditions (Table 1). In the cases of **1** and **2,** negligible reactivity was observed (Table 1, entries 1 and 2), while with **3,** excellent conversion to the desired product was achieved. The drastic increase in reactivity may be addressed to the increased steric demand of the ligand in comparison to complexes **1** and **2**. The importance of the steric demand of the bisphosphine ligand for the reactivity of alkyl complexes was also demonstrated previously for the hydrogenation of alkenes.^13^ The stability of the active species may be preserved due to increased steric hindrance. It should be noted that the hydrogenation of ketones at room temperature is comparingly rare in the field of manganese(I) chemistry.^4f,4g^ So far, Mn(I)-catalyzed base-free hydrogenation reactions are only known for aldehydes,^3a^ nitriles,^9a^ N-heterocycles,^11b,11c^ and alkenes.^13^

**Table 1:**
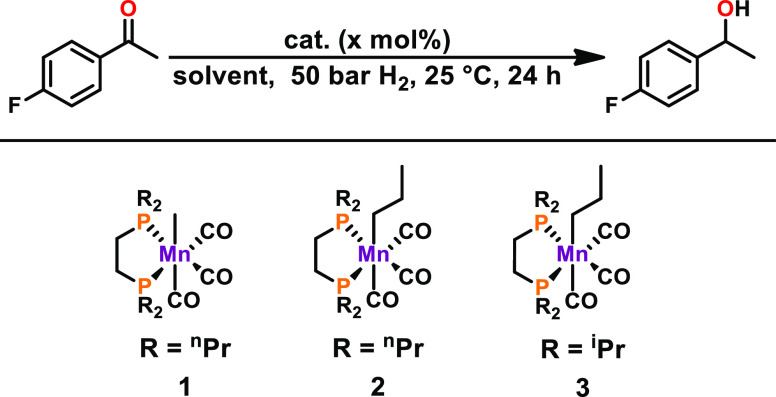
Optimization Reaction for the Hydrogenation of 4-Fluoroacetophenone^a^

entry	catalyst (mol %)	solvent	conversion (%)
1	**1** (3)	Et_2_O	
2	**2** (3)	Et_2_O	traces
3	**3** (3)	Et_2_O	95
4	**3** (3)	MeOH	31
5	**3** (3)	DCM	30
6	**3** (3)	THF	69
7	**3** (3)	DME	83
8^b^	**3** (3)	Et_2_O	>99
**9****^c^**	**3** (3)	**Et**_**2**_**O**	**>99**
10^c^	**3** (2)	Et_2_O	69
11^c^^,^^d^	**3** (3)	Et_2_O	22

a Reaction conditions: 4-fluoroacetophenone (0.38 mmol), 5 mL anhydrous solvent, 25 °C, 50 bar H_2_, 24 h, conversion determined *via*^19^F{^1^H}-NMR spectroscopy.

b 30 bar H_2_.

c 10 bar H_2_.

d 8 h.

In other solvents such as MeOH, CH_2_Cl_2_, or dimethoxyethane (DME), lower reactivities were observed. Interestingly, lowering the hydrogen pressure from 50 to 10 bar resulted in full conversion (Table 1, entry 9), which is comparatively low for manganese-based catalysts. A shorter reaction time (8 h) led to a drastic decrease in conversion (Table 1, entry 11), which might be attributed to an induction period required for catalyst activation.

Having determined **3** as the most active catalyst and to prove its general applicability, various substrates have been tested to establish scope and limitations (Table 2). The catalytic experiments were conducted in the presence of 3 mol % of catalyst at 25 °C and 10 bar hydrogen pressure, a reaction time of 24 h, without the addition of any additives. Within this context, halide-containing substrates (Table 2, entries **4–7**) as well as substrates with electron-donating groups (Table 2, entries **11** and **12**) gave excellent yields. Lower reactivity could be detected for substrates containing a coordinating amine or pyridine (Table 2, entries **13** and **19**). No conversion could be detected for substrate **9**, bearing the strongly coordinating nitrile functionality. Furthermore, no reaction was observed in the presence of a nitro group (Table 2, entry **10**), presumably due to the possible undesired redox reactions with the catalyst. In the case of sterically more demanding substrate **15,** only a moderate conversion could be achieved. Aliphatic ketones were very efficiently reduced to the corresponding alcohols (Table 2, entries **21–23**). However, the reaction time had to be increased to achieve high conversions. Manganese-catalyzed hydrogenations of ketones at room temperature are relatively rare,^4f,4g^ and to the best of our knowledge, an additive-free hydrogenation of ketones has not been reported.

**Table 2:**
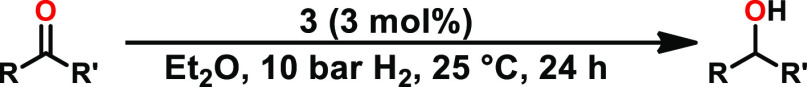

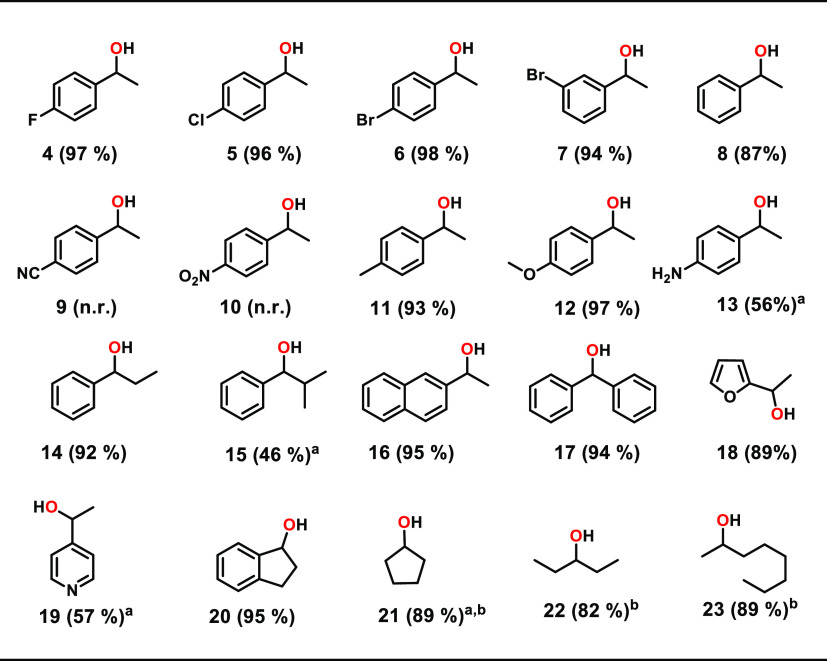
Scope and Limitation for the Hydrogenation of Ketones Catalyzed by **3**^a^

a Reaction conditions: ketone (0.38 mmol), **3** (3 mol %), 5 mL anhydrous Et_2_O, 10 bar H_2_, 25 °C, 24 h; isolated yields.

b Conversion determined *via* GC–MS.

c 36 h.

Furthermore, a potential temperature-dependent selectivity for the hydrogenation of α,β-unsaturated carbonyls was investigated (Table 3). At room temperature, the high selectivity for the reduction of the carbonyl group could be detected, whereas the C=C bond stays unaltered (Table 3, **24–27**). Interestingly, if hydrogenation was carried out at 60 °C, fully saturated systems (Table 3, **28–30**) were received as products. Additionally, the catalyst loading could be decreased to 1 mol %. The reaction barrier for the hydrogenation of 1,2-disubstituted C–C double bonds is generally higher than for ketones, requiring a higher reaction temperature, as demonstrated previously.^13^ In the case of citral as the substrate, solely the C=O and not the trisubstituted C=C bond was hydrogenated (Table 3, **25**). This temperature-dependent selectivity for the reduction of α,β-unsaturated carbonyl moieties may be interesting for synthetic applications.

**Table 3:**
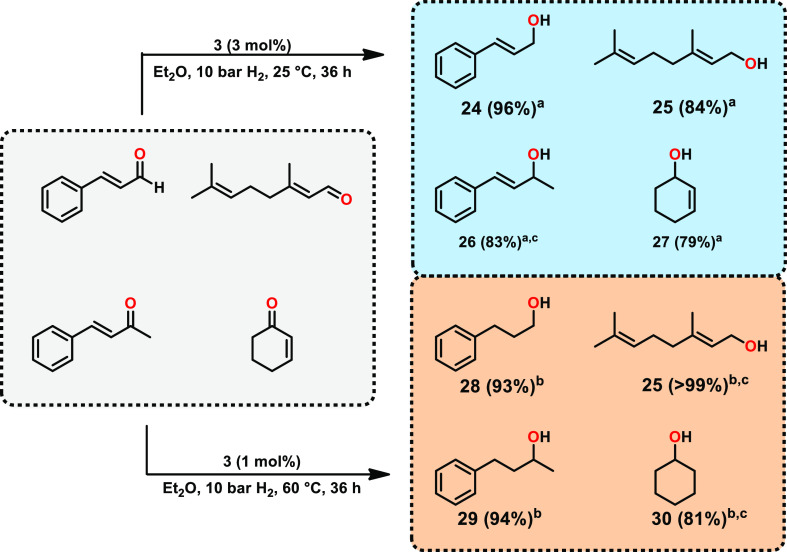
Temperature Dependence of the Hydrogenation of α,β-Unsaturated Carbonyls Catalyzed by **3**

a Reaction conditions: ketone (0.38 mmol), **3** (3 mol %), 5 mL anhydrous Et_2_O, 10 bar H_2_, 25 °C, 36 h; isolated yields.

b Ketone (0.38 mmol), **3** (1 mol %), 5 mL anhydrous Et_2_O, 10 bar H_2_, 60 °C, 36 h; isolated yields.

c Conversion determined *via* GC–MS.

A mechanistic investigation of the introduced system revealed that the reactivity of **3** was drastically lowered upon the addition of 1 equiv of PMe_3_ (with 4-fluoroacetophenone as the substrate). This finding indicates the presence of an inner-sphere reaction, as the strong donor PMe_3_ apparently blocks the vacant coordination site of the active catalyst for the incoming substrates. The homogeneity of the system was proven by the Hg drop test as no significant decrease in reactivity could be detected.

**Figure 1:**
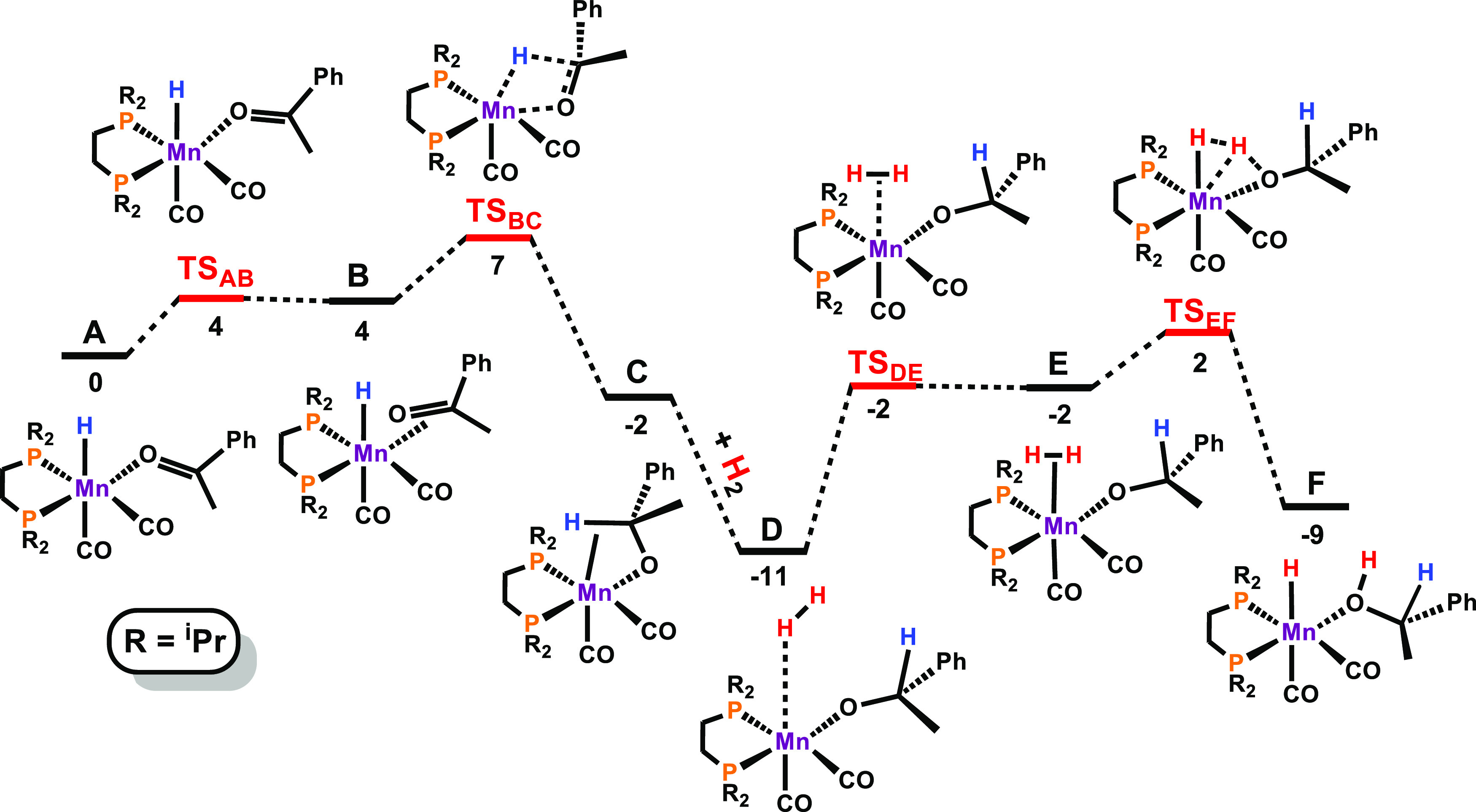
Free-energy profile calculated for the hydrogenation of acetophenone. Free energies (kcal/mol) are referred to intermediate **A**.

**Scheme 3:**
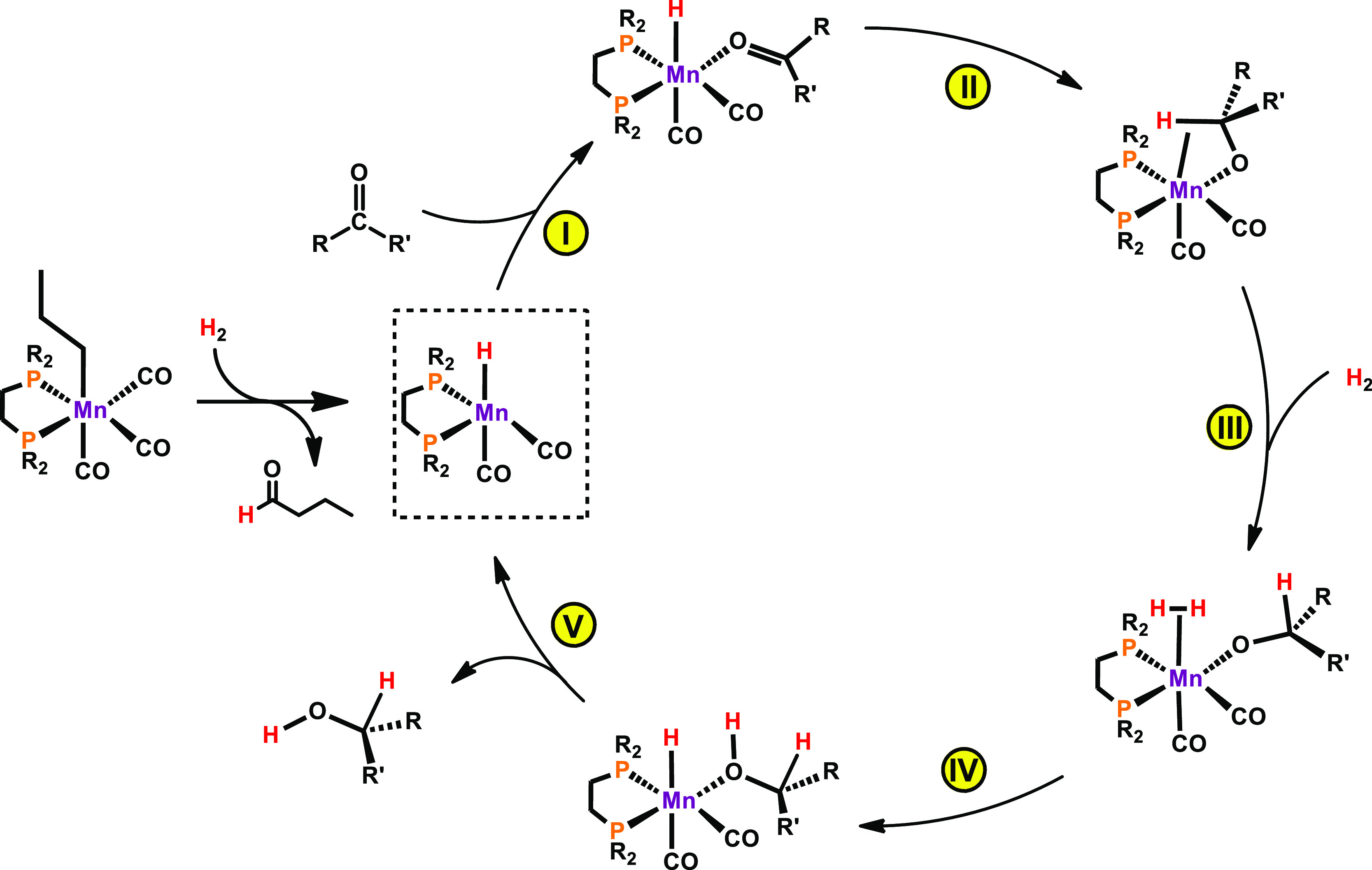
Simplified Catalytic Cycle for the Hydrogenation of Ketones

The mechanism of hydrogenation of ketones by **3** was investigated in detail by DFT calculations using acetophenone as the model substrate. The resulting free-energy profile is represented in Figure 1 while Scheme 3 depicts a summary of the catalytic cycle.

Catalyst initiation, starting from **3**, has been reported previously.^13^ Acetophenone coordination to the 16-electron hydride intermediate forms intermediate **A**, a κ^1^-(O) complex that rearranges to a η^2^-coordination mode in **B**. This is a facile process with a barrier of only 4 kcal/mol (**TS**_**AB**_). From **B**, there occurs an attack of the hydride on the carbonyl C atom, resulting in **C**, an alkoxide complex stabilized by an agostic interaction involving the recently formed C–H bond. The formation of **C**, from **B**, is also easy with a barrier of only 3 kcal/mol (**TS**_**BC**_), being a favorable step, from the thermodynamic point of view with Δ*G* = −6 kcal/mol. The path proceeds with the dihydrogen addition to the alkoxide intermediate, from **D** to **E**, overcoming a barrier of 9 kcal/mol, measured from the pair of molecules (H_2_ + alkoxide intermediate) in **D** to **TS**_**DE**_. This is an endergonic step with Δ*G* = 9 kcal/mol. Finally, in the last step of the cycle, there occurs H transfer from the H_2_ ligand to the alkoxide O atom, regenerating the hydride and forming the O-coordinated alcohol product in **F**. This is a clearly favorable process (Δ*G* = −7 kcal/mol) with a barrier of 4 kcal/mol (**TS**_**EF**_), from **E** to **F**. The cycle is closed by the release of the product (1-phenylethanol) and the coordination of a new acetophenone molecule, from **F** back to **A**, a process with a free energy balance of 5 kcal/mol. The least stable transition state is the one associated with the hydride attack on the carbonyl C atom (**TS**_**BC**_), and the overall barrier for the catalytic cycle is 14 kcal/mol, measured from the most stable intermediate (**D**) to **TS**_**BC**_ of the following cycle.

## Conclusions

In conclusion, the hydrogenation of aromatic and aliphatic ketones using a bench-stable Mn(I) alkyl complex is described. The reaction proceeds under mild conditions (10 bar H_2_, 25 °C) and notably without the addition of any additives. Under these conditions, chemoselective hydrogenation of the carbonyl moiety of α,β-unsaturated carbonyls could be achieved. Interestingly, if the reaction was carried out at 60 °C, 1,2-disubstituted C=C bonds are additionally reduced, whereas a trisubstituted C=C bond stays intact. A detailed reaction mechanism based on DFT calculations is presented. The precatalyst is activated by dihydrogen upon the migratory insertion of the alkyl group into the adjacent CO ligand and consecutive split of the coordinated dihydrogen. The catalytic reaction proceeds *via* an inner-sphere reaction upon substrate coordination, insertion, dihydrogen activation, and regeneration of the active species due to product release.

## Experimental Section

### General Information

All reactions were performed under an inert atmosphere of argon using Schlenk techniques or in a MBraun inert gas glovebox. The solvents were purified according to standard procedures. The deuterated solvents were purchased from Aldrich and dried over 3 Å molecular sieves. Complexes *fac*-[Mn(dpre) (CO)_3_(Me)] (dpre = 1,2-*bis*(di-*n*-propylphosphino)ethane) (**1**), *fac*-[Mn(dpre) (CO)_3_(Pr)] (**2**), and *fac*-[Mn(dippe) (CO)_3_(Pr)] (dippe = 1,2-*bis*(di-*iso*-propylphosphino)ethane) (**3**) were synthesized according to the literature.^13^^1^H- and ^13^C{^1^H}-NMR spectra were recorded on Bruker AVANCE-250 and AVANCE-400 spectrometers. ^1^H and ^13^C{^1^H}-NMR spectra were referenced internally to residual protio-solvent and solvent resonances, respectively, and are reported relative to tetramethylsilane (δ = 0 ppm). Hydrogenation reactions were carried out in a Roth steel autoclave using a Tecsis manometer. GC–MS analysis was conducted on an ISQ LT single quadrupole MS system (Thermo Fisher) directly interfaced to a TRACE 1300 gas chromatographic system (Thermo Fisher), using a Rxi-5Sil MS (30 m, 0.25 mm ID) cross-bonded dimethyl polysiloxane capillary column.

### General Procedure for the Hydrogenation of Ketones

Inside an Ar-flushed glovebox, ketone substrate (0.38 mmol, 1 equiv) and **3** (3 mol %) were dissolved in 5 mL of Et_2_O and taken up in a syringe. The mixture was injected into a steel autoclave, and the reaction vessel was flushed three times with 10 bar H_2_. The reaction was stirred for the indicated time. The autoclave was depressurized and the sample was taken for GC–MS analysis. The reaction mixture was passed through a pad of silica. The silica pad was rinsed with Et_2_O, and the solvent was gently removed.

### Computational Details

The computational results presented have been achieved in part using the Vienna scientific cluster. All calculations were performed using the Gaussian 09 software package.^14^ Geometry optimizations were obtained using the Perdew, Burke, and Ernzerhof (PBE)0 functional without symmetry constraints, a basis set consisting of the Stuttgart/Dresden ECP basis set^15^ to describe the electrons of Mn, and a standard 6-31G(d,p) basis set^16^ for all other atoms. The PBE0 functional uses a hybrid generalized gradient approximation, including 25% mixture of Hartree–Fock^17^ exchange with DFT^18^ exchange–correlation, obtained by the PBE functional.^19^ Transition-state optimizations were performed with the synchronous transit-guided quasi-Newton method developed by Schlegel *et al.*,^20^ following extensive searches of the potential energy surface. Frequency calculations were performed to confirm the nature of the stationary points, yielding one imaginary frequency for the transition states and none for the minima. Each transition state was further confirmed by following its vibrational mode downhill on both sides and obtaining the minima presented on the energy profiles. The electronic energies were converted to free energy at 298.15 K and 1 atm using zero-point energy and thermal energy corrections based on the structural and vibration frequency data calculated at the same level. The free-energy values presented were corrected for dispersion by means of the Grimme DFT-D3 method,^21^ with the Becke and Johnson short-distance damping.^22^ Solvent effects (Et_2_O) were considered in all the calculations using the polarizable continuum model initially devised by Tomasi and co-workers,^23^ with the radii and nonelectrostatic terms of the SMD solvation model developed by Truhlar *et al.*(24)

## Supporting Information Available

^1^H NMR and ^13^C{^1^H} NMR spectra of all compounds (PDF)
Cartesian coordinates for DFT-optimized structures (XYZ)

## Supplementary Material

om1c00161_si_001.pdf

om1c00161_si_002.xyz
