# Supplementary material for: Manganese-Catalyzed Hydrogenation of Ketones under Mild and Base-free Conditions
Source: Organometallics. 2021 Apr 22;40(9):1388–94. doi: 10.1021/acs.organomet.1c00161 (PMC8155567; doi:10.1021/acs.organomet.1c00161)
Supplement: Supplementary file 1 — om1c00161_si_001.pdf [file om1c00161_si_001.pdf]

Supporting Information

Manganese-Catalyzed Hydrogenation of Ketones under Mild and Base-free Conditions

Stefan Weber,[†] Juilan Brünig,[†] Luis F. Veiros,[§] and Karl Kirchner*[, †]

[†]Institute of Applied Synthetic Chemistry, Vienna University of Technology, Getreidemarkt 9, A-1060 Vienna, AUSTRIA

[§] Centro de Química Estrutural and Departamento de Engenharia Química, Instituto Superior Técnico, Universidade de Lisboa, Av Rovisco Pais, 1049-001 Lisboa, PORTUGAL

E-mail: karl.kirchner@tuwien.ac.at

1. Characterization of organic products 2
2. NMR spectra of all isolated products 6
3. Reference 25

1. Characterization of organic products

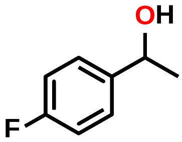

1-(4-Fluorophenyl)ethanol[1] (**4**), colorless liquid (97 %). ^{1}H NMR (δ, 400 MHz, CD_2Cl_2, 20 °C): 7.42 – 7.36 (m, 2H), 7.11 – 7.03 (m, 2H), 4.91 (qd, J = 6.5, 3.8 Hz, 1H), 1.99 (s, 1H), 1.48 (d, J = 6.4 Hz, 3H). ^{13}C{^{1}H} NMR (δ, 101 MHz, CD_2Cl_2, 20 °C): 162.0 (d, J_{CF} = 244.0 Hz), 142.0 (d, J_{CF} = 3.0 Hz), 127.1 (d, J_{CF} = 7.9 Hz), 115.0 (d, J_{CF} = 21.2 Hz), 69.5, 25.2.

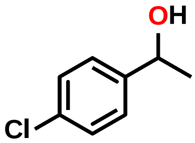

1-(4-Chlorophenyl)ethanol[1] (**5**), slightly yellow liquid (96 %). ^{1}H NMR (δ, 250 MHz, C_6D_6, 20 °C):7.42 – 7.29 (m, 1H), 7.06 (d, J = 4.2 Hz, 1H), 6.88 (dd, J = 15.8, 8.5 Hz, 1H), 4.30 (q, J = 6.5 Hz, 1H), 1.88 (s, 1H), 1.09 (d, J = 6.4 Hz, 3H). ^{13}C{^{1}H} NMR (δ, 63 MHz, C_6D_6, 20 °C): 144.7, 129.5, 128.3, 126.3, 69.1, 25.0.

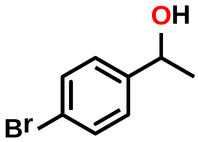

1-(4-Bromophenyl)ethanol[2] (**6**), yellow oil (98 %). ^{1}H NMR (400 MHz, $CDCl_3$, 20°C) δ = 7.47 (d, J = 7.9 Hz, 2H), 7.24 (d, J = 8.0 Hz, 2H), 5.01 – 4.69 (m, 1H), 2.17 (s, 1H), 1.49 (d, J = 5.5 Hz, 3H). ^{13}C{^{1}H}NMR (101 MHz, $CDCl_3$, 20°C) δ = 144.8 , 131.5 , 127.2 , 121.1 , 69.8 , 25.3.

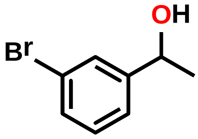

1-(3-Bromophenyl)ethanol[3] (**7**), yellow oil (94 %). ^{1}H NMR (δ, 250 MHz, C_6D_6, 20 °C): δ = 7.38-7.34 (m, 1H), 7.14 – 7.07 (m, 1H), 6.86 (s, 1H), 6.70 (t, J = 7.8 Hz, 1H), 4.21 (q, J = 6.4 Hz, 1H), 1.13 (s, 1H), 1.03 (d, J = 6.4 Hz, 4H). ^{13}C{^{1}H} NMR (δ, 63 MHz, C_6D_6, 20 °C): δ = 148.8, 130.0, 129.8, 128.6, 123.8, 122.5, 69.0, 25.0.

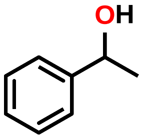

1-Phenylethanol[1] (**8**), slightly yellow liquid (87 %). ^{1}H NMR (400 MHz, $CDCl_3$, 20°C) δ = 7.36 – 7.29 (m, 4H), 7.27 – 7.21 (m, 1H), 4.86 (q, J = 6.5 Hz, 1H), 1.75 (s, 1H), 1.46 (d, J = 6.5 Hz, 3H). ^{13}C {^{1}H} NMR (101 MHz, $CDCl_3$, 20°C) δ = 148.6, 130.3, 128.0, 125.1, 71.3, 26.7.

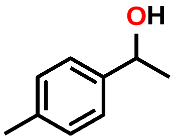

1-(*p*-Tolyl)ethanol[1] (**11**), yellow oil (93%). ^{1}H (400 MHz, $CDCl_3$, 20°C) δ = 7.55 – 6.99 (m, 4H), 4.86 (m, 1H), 2.60 (s, 1H), 2.41 (s, 3H), 1.76 – 1.57 (m, 3H). ^{13}C {^{1}H} NMR (101 MHz, $CDCl_3$, 20°C) δ = 143.5, 138.3, 130.5, 126.8, 70.0, 27.2, 22.4.

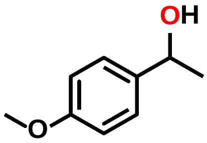

1-(4-Methoxyphenyl)ethanol[1] (**12**), yellow oil (97%). ^{1}H NMR (400 MHz, $CDCl_3$, 20°C) δ = 7.19 (d, J = 8.2 Hz, 2H), 6.77 (d, J = 8.0 Hz, 2H), 4.74 (q, J = 6.2 Hz, 1H), 3.69 (s, 4H), 2.01 (s, 1H), 1.37 (d, J = 5.1 Hz, 3H). ^{13}C {^{1}H} NMR (101 MHz, $CDCl_3$, 20°C) δ = 158.8, 138.0, 126.6, 113.7, 69.9, 55.2, 25.0.

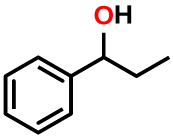

1-Phenylpropanol[2] (**14**), colorless liquid (92 %). ^{1}H NMR (400 MHz, CD_2Cl_2, 20°C) δ = 7.53 – 7.39 (m, 4H), 7.34 – 7.27 (m, 1H), 4.62 (ddd, J = 7.2, 6.0, 3.6 Hz, 1H), 1.96 (d, J = 3.6 Hz, 1H), 1.89 – 1.69 (m, 2H), 0.94 (t, J = 7.4 Hz, 3H). ^{13}C {^{1}H} NMR (101 MHz, CD_2Cl_2, 20°C) δ = 145.0, 128.3, 127.3, 125.9, 75.7, 32.1, 9.9.

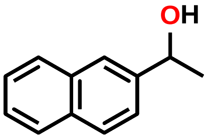

1-Naphtalen-2-yl-ethanol[1] (**16**), colorless solid (95 %). ^{1}H NMR (400 MHz, $CDCl_3$, 20°C) δ = 7.98 – 7.71 (m, 4H), 7.56 – 7.34 (m, 3H), 5.18 – 4.86 (m, 1H), 2.62 (s, 1H), 1.57 (d, J = 6.0 Hz, 3H). ^{13}C {^{1}H} NMR (101 MHz, $CDCl_3$, 20°C) δ = 144.2, 134.3, 133.8, 129.2, 128.89, 128.6, 127.1, 126.7, 124.8, 124.7, 71.4, 26.1.

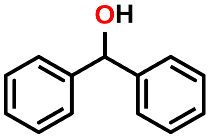

Diphenylmethanol[1] (**17**), colorless solid (94 %). ^{1}H NMR (400 MHz, $CDCl_3$, 20°C) δ = 7.57 – 7.19 (m, 10H), 5.86 (s, 1H), 2.42 (s, 1H). ^{13}C {^{1}H} NMR (101 MHz, $CDCl_3$, 20°C) δ = 144.6, 129.3, 128.4, 127.4, 77.1.

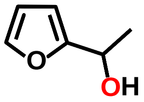

1-(furan-2-yl)ethanol[1] (**18**), colorless oil (89 %). ^{1}H NMR (400 MHz, $CDCl_3$, 20°C) δ = 7.41 – 7.32 (m, 1H), 6.35 – 6.29 (m, 1H), 6.24 – 6.17 (m, 1H), 4.87 (q, J = 6.7, 5.3 Hz, 1H), 2.10 (s, 1H), 1.54 (d, J = 6.6 Hz, 3H). ^{13}C {^{1}H} NMR (101 MHz, $CDCl_3$, 20°C) δ = 157.1, 141.4, 109.6, 104.6, 63.1, 20.7.

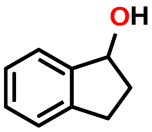 2,3-dihydro-1*H*-inden-1-ol[2] (**20**), colorless solid (95 %). ^{1}H NMR (400 MHz, $CDCl_3$, 20°C) δ = 7.45 – 7.34 (m, 1H), 7.28 – 7.19 (m, 3H), 5.23 – 5.17 (m, 1H), 3.07 – 2.99 (m, 1H), 2.85 – 2.73 (m, 1H), 2.50 – 2.39 (m, 1H), 2.15 (s, 1H), 1.97 – 1.86 (m, 1H). ^{13}C {^{1}H} NMR (101 MHz, $CDCl_3$, 20°C) δ = 146.2, 144.5, 129.5, 127.9, 126.1, 125.4, 77.6, 37.1, 31.0.

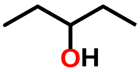 3-Pentanol[4] (**22**), colorless liquid (82 %). ^{1}H (400 MHz, C_6D_6, 20°C) δ = 3.50 (s, 1H), 3.35 (p, J = 6.0 Hz, 1H), 1.48 – 1.38 (m, 4H), 0.94 (t, J = 7.8 Hz, 6H). ^{13}C {^{1}H} NMR (101 MHz, C_6D_6, 20°C) δ = 72.6, 28.2, 8.4.

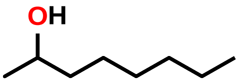 2-Octanol[4] (**23**) colorless liquid (89 %). ^{1}H (400 MHz, CD_2Cl_2, 20°C) δ = 3.56 - 3.50 (m, 2H), 1.42 – 1.23 (m, 10H), 0.94 – 0.85 (m, 6H). ^{13}C {^{1}H} NMR (101 MHz, CD_2Cl_2, 20°C) δ = 65.0, 42.0, 30.1, 29.1, 23.3, 23.1, 13.8, 10.9.

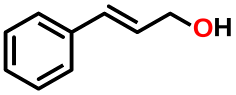 *E*-3-phenylprop-2-enol[2] (**24**) colorless oil (96 %). ^{1}H (400 MHz, CD_2Cl_2, 20°C) δ = 7.34 – 7.29 (m, 2H), 7.23 (ddd, J = 7.8, 6.2, 1.3 Hz, 2H), 7.18 – 7.12 (m, 1H), 6.52 (dt, J = 15.9, 1.7 Hz, 1H), 6.28 (dt, J = 16.0, 5.6 Hz, 1H), 4.19 (dd, J = 5.6, 1.6 Hz, 2H), 1.61 (s, 1H). ^{13}C {^{1}H} NMR (101 MHz, CD_2Cl_2, 20°C) δ =136.9, 130.5, 129.0, 128. 6, 127.6, 126.4, 63.4.

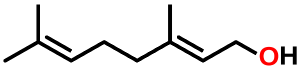 *E*-3,7-dimethylocta-2,6-dienol[5] (**25**) slightly yellow oil (84 %). ^{1}H (400 MHz, CD_2Cl_2, 20°C) δ = 5.40 (ddq, *J* = 6.9, 5.4, 1.3 Hz, 1H), 5.12 (tdq, *J* = 7.2, 2.9, 1.4 Hz, 1H), 4.21 – 4.02 (m, 1H), 2.28 – 1.94 (m, 4H), 1.78 - 1.69 (m, 5H), 1.62 (s, 3H), 1.39 – 1.05 (m, 2H). ^{13}C {^{1}H} NMR (101 MHz, CD_2Cl_2, 20°C) δ = 139.2, 131.6, 123.9, 123.6, 59.2, 39.5, 26.4, 25.4, 17.4, 15.9.

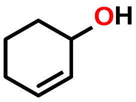 Cylohex-2-en-ol[6] (**27**) slightly yellow liquid (79 %). ^{1}H NMR (δ, 250 MHz, C_6D_6, 20 °C): δ = 5.98 – 5.79 (m, 1H), 5.77 – 5.58 (m, 1H), 4.23 - 4.09 (m, 1H), 3.92 (s, 2H), 2.04 – 1.51 (m, 8H), 1.50 – 1.20 (m, 2H). ^{13}C{^{1}H} NMR (δ, 63 MHz, C_6D_6, 20 °C): δ = 130.8, 129.1, 65.2, 32.0, 25.0, 19.3.

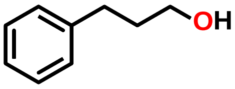

3-Phenylpropanol[7] (**28**) slightly yellow oil (93 %). ^{1}H NMR (δ, 400 MHz, C_6D_6, 20 °C): δ = 7.18 – 7.11 (m, 2H), 7.08 – 6.99 (m, 3H), 3.37 (t, J = 6.5 Hz, 2H), 2.53 (t, J = 7.6 Hz, 2H), 1.95 (s, 1H), 1.68 (t, J = 7.3 Hz, 2H). ^{13}C {^{1}H} NMR (101 MHz, C_6D_6, 20°C) δ = 142.8, 129.2, 129.1, 126.5, 62.4, 35.1, 32.8.

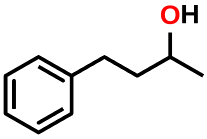

4-Phenylbutan-2-ol[7] (**29**) colorless oil (91 %). ^{1}H (400 MHz, C_6D_6, 20°C) δ = 7.44 -6.96(m, 5H), 3.65- 3.46 (m, 1H), 2.68 – 1.85 (m, 2H), 1.78 – 1.42 (m, 3H), 1.22 -0.89 (m, 3H). ^{13}C {^{1}H} NMR (101 MHz, C_6D_6, 20°C) δ = 142.3, 128.4, 128.3, 125.7, 66.6, 40.9, 32.1, 23.4.

2. NMR spectra of all isolated products

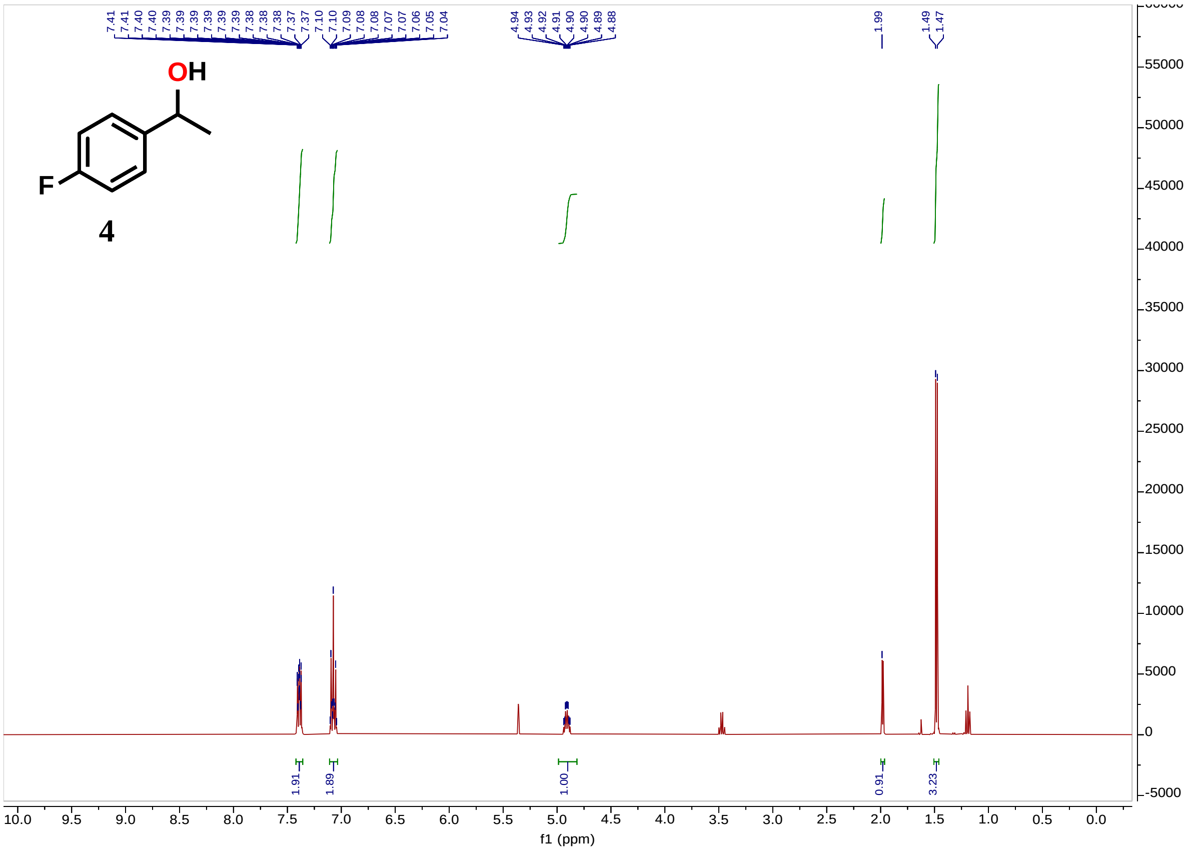

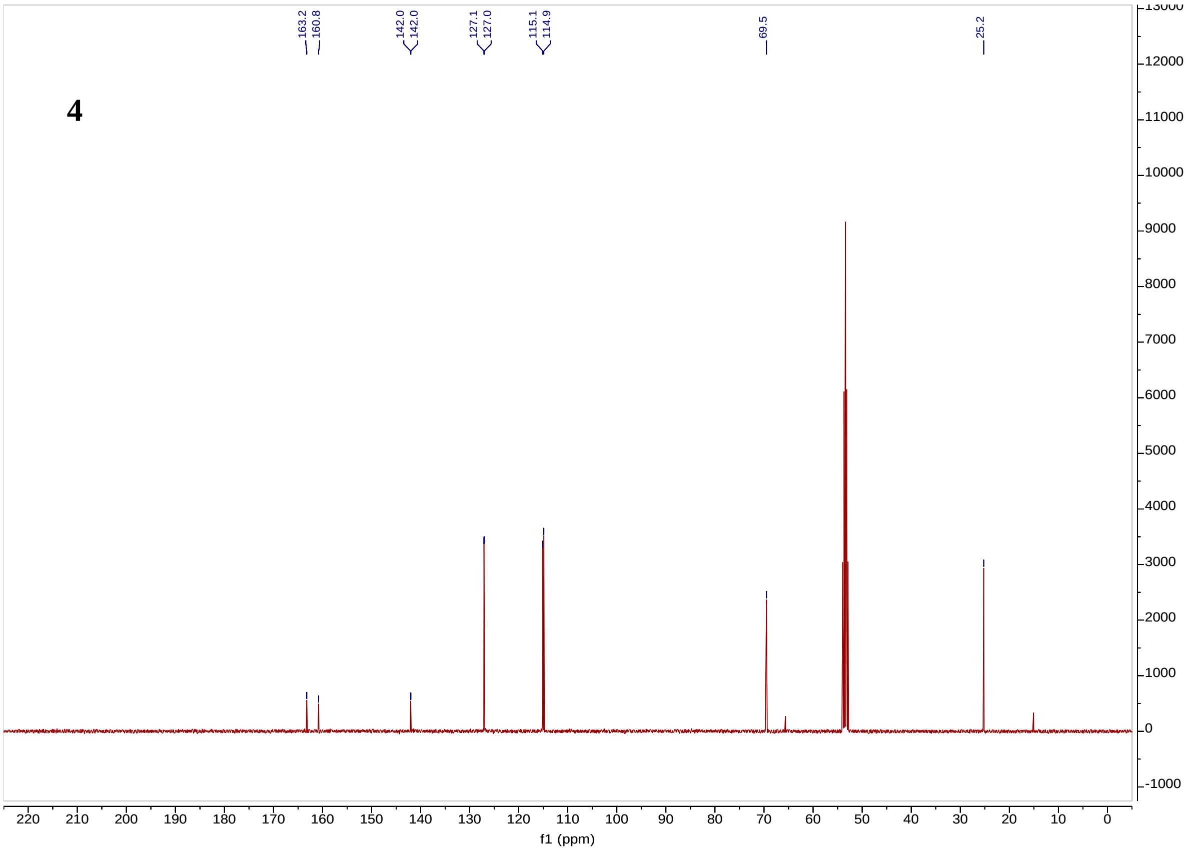

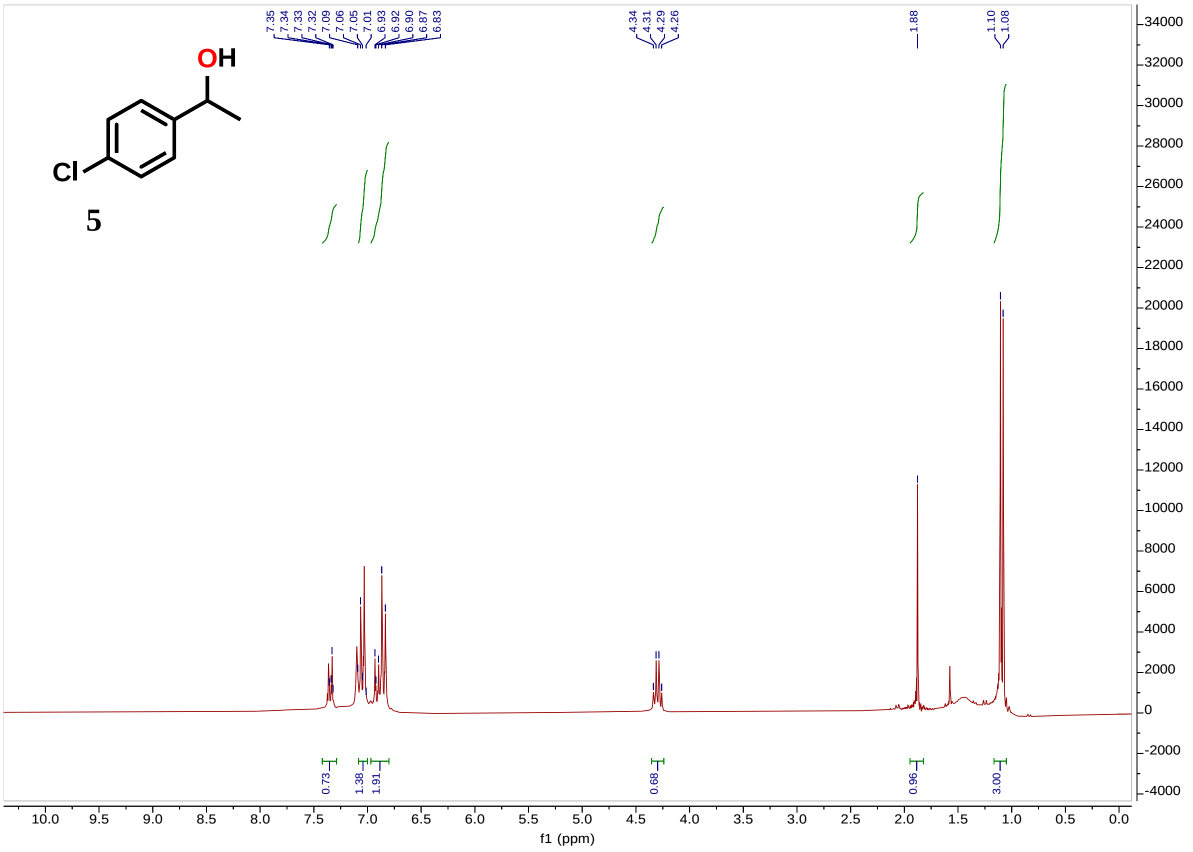
7.35 7.34 7.33 7.32 7.09 7.06 7.05 7.01 6.93 6.92 6.90 6.87 6.83
4.34 4.31 4.29 4.26
1.88
1.10 1.08
OH
Cl
5
0.73
1.38
1.91
0.68
0.96
3.00
f1 (ppm)

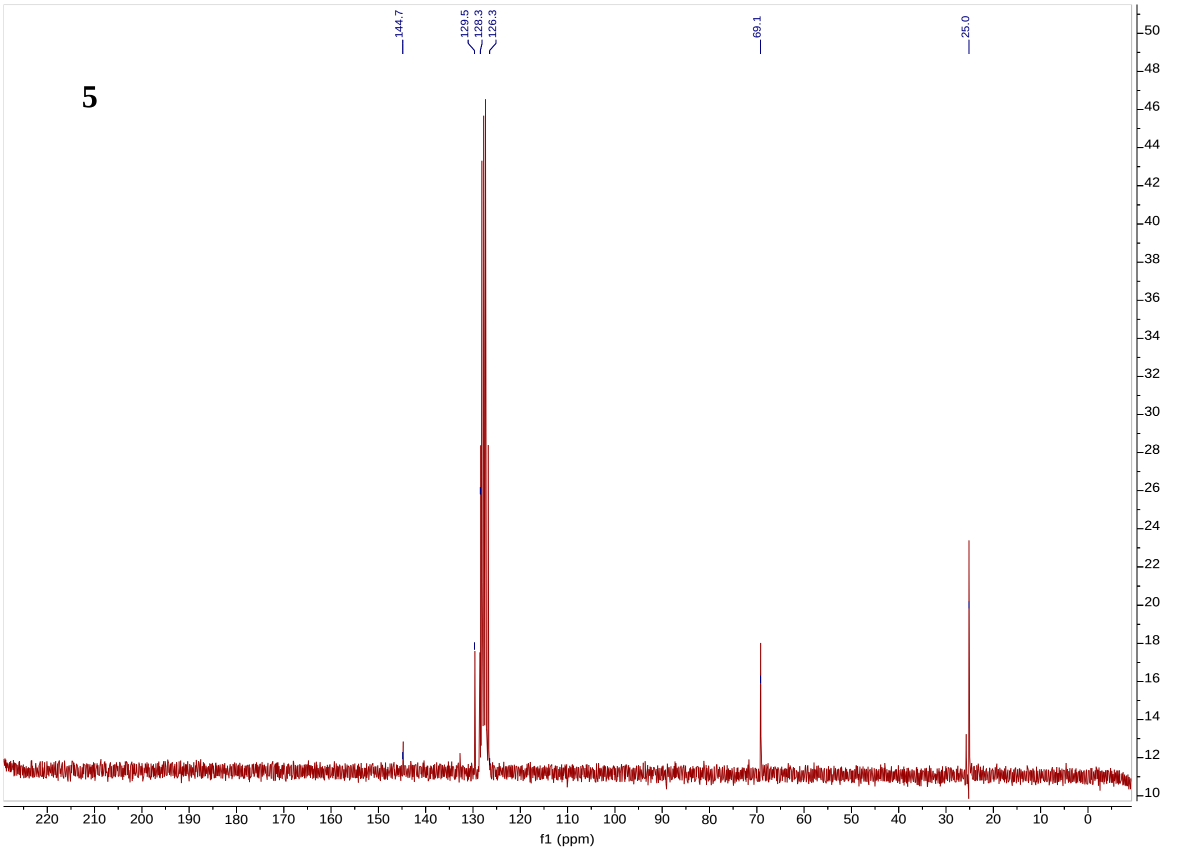
144.7
129.5 128.3 126.3
69.1
25.0
5
f1 (ppm)

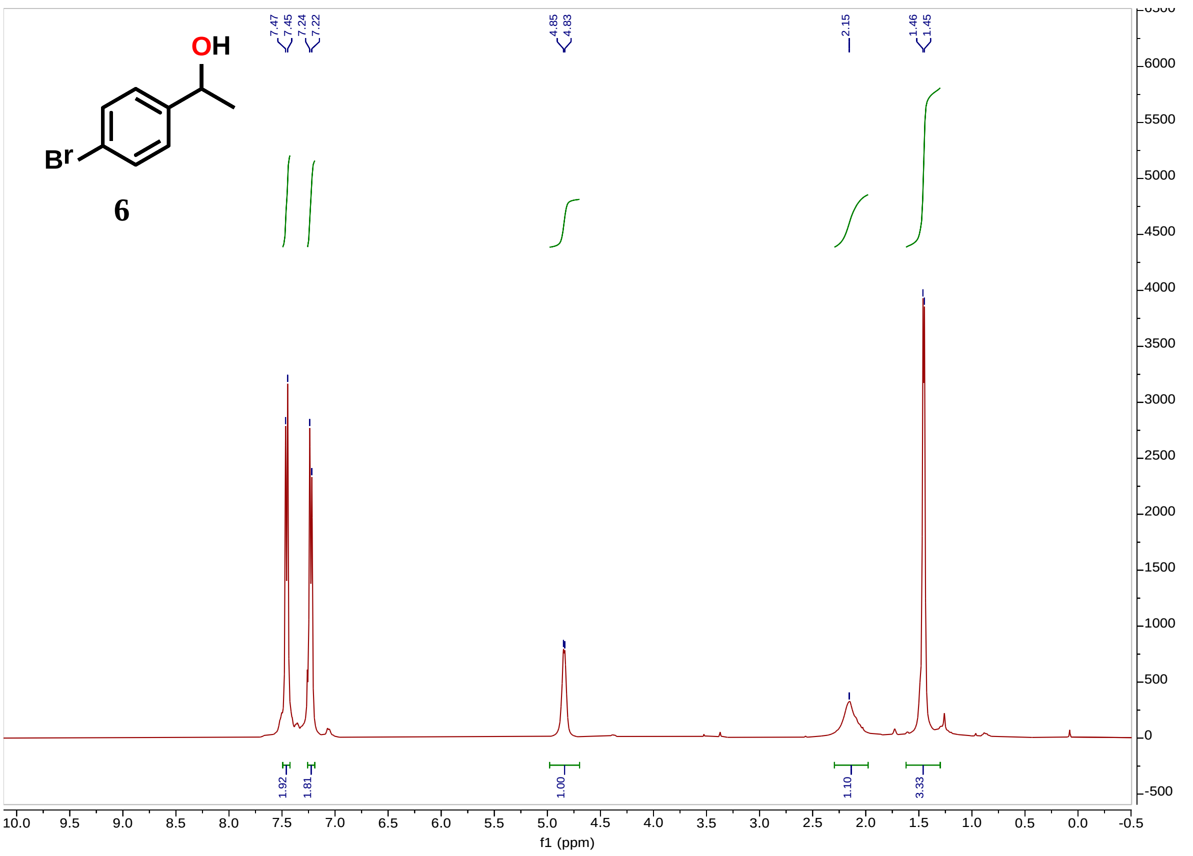
OH
Br
6
7.47
7.45
7.24
7.22
4.85
4.83
2.15
1.46
1.45
1.92
1.81
1.00
1.10
3.33
f1 (ppm)

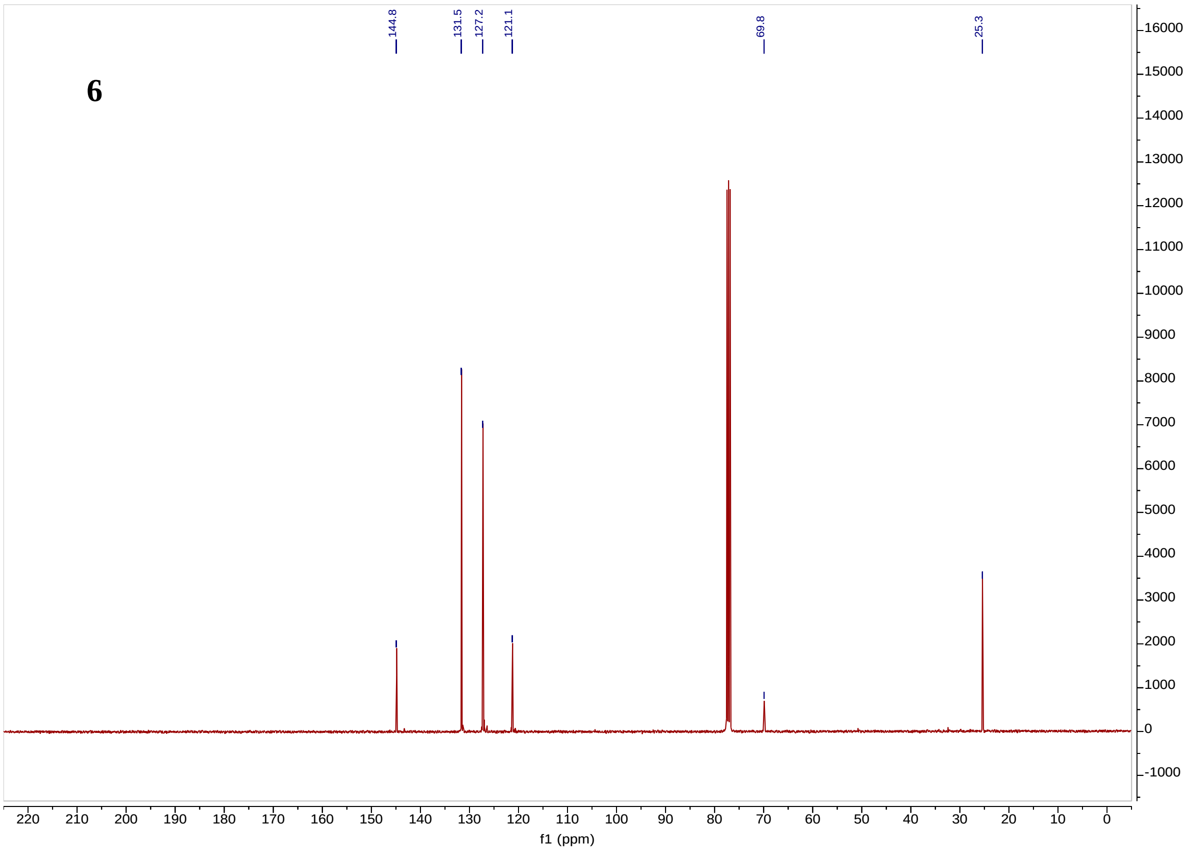
6
144.8
131.5
127.2
121.1
69.8
25.3
f1 (ppm)

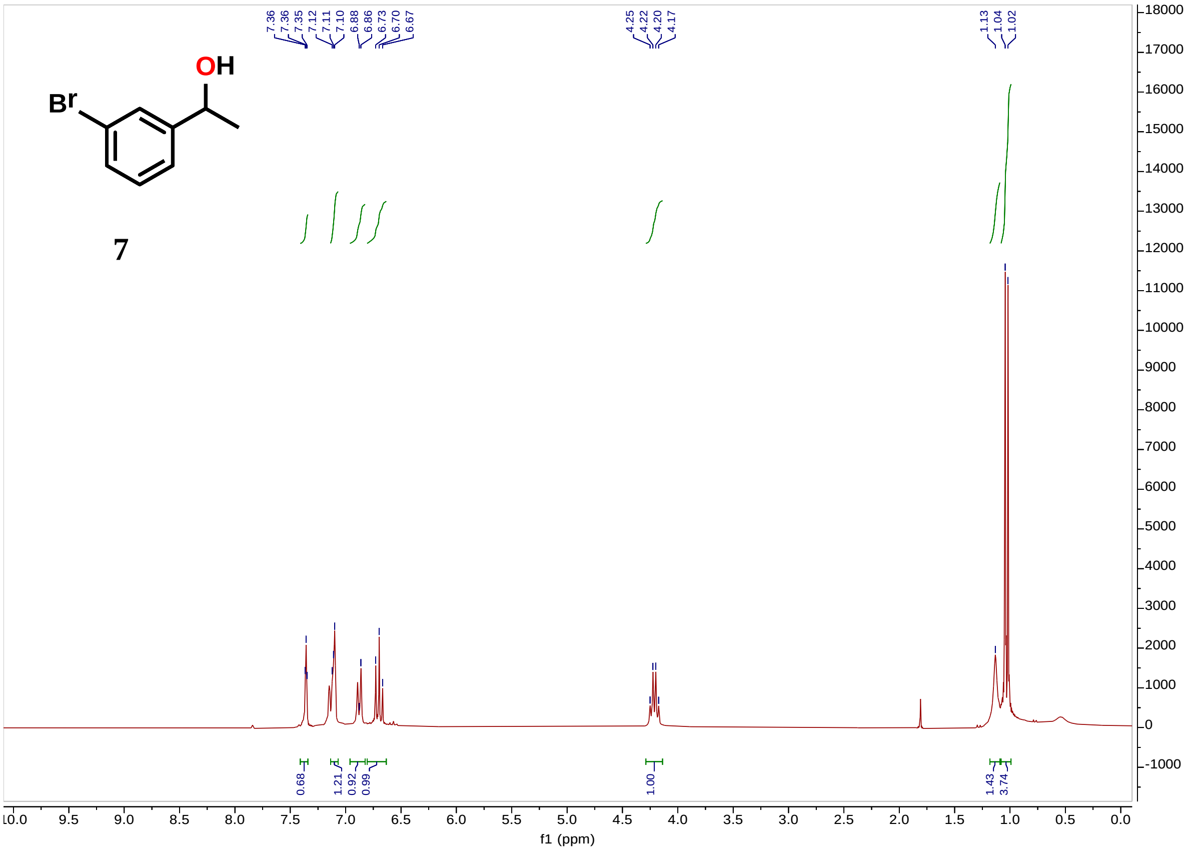
7.36
7.36
7.35
7.12
7.11
7.10
6.88
6.86
6.73
6.70
6.67
4.25
4.22
4.20
4.17
1.13
1.04
1.02
Br
OH
7
0.68
1.21
0.92
0.99
1.00
1.43
3.74
f1 (ppm)

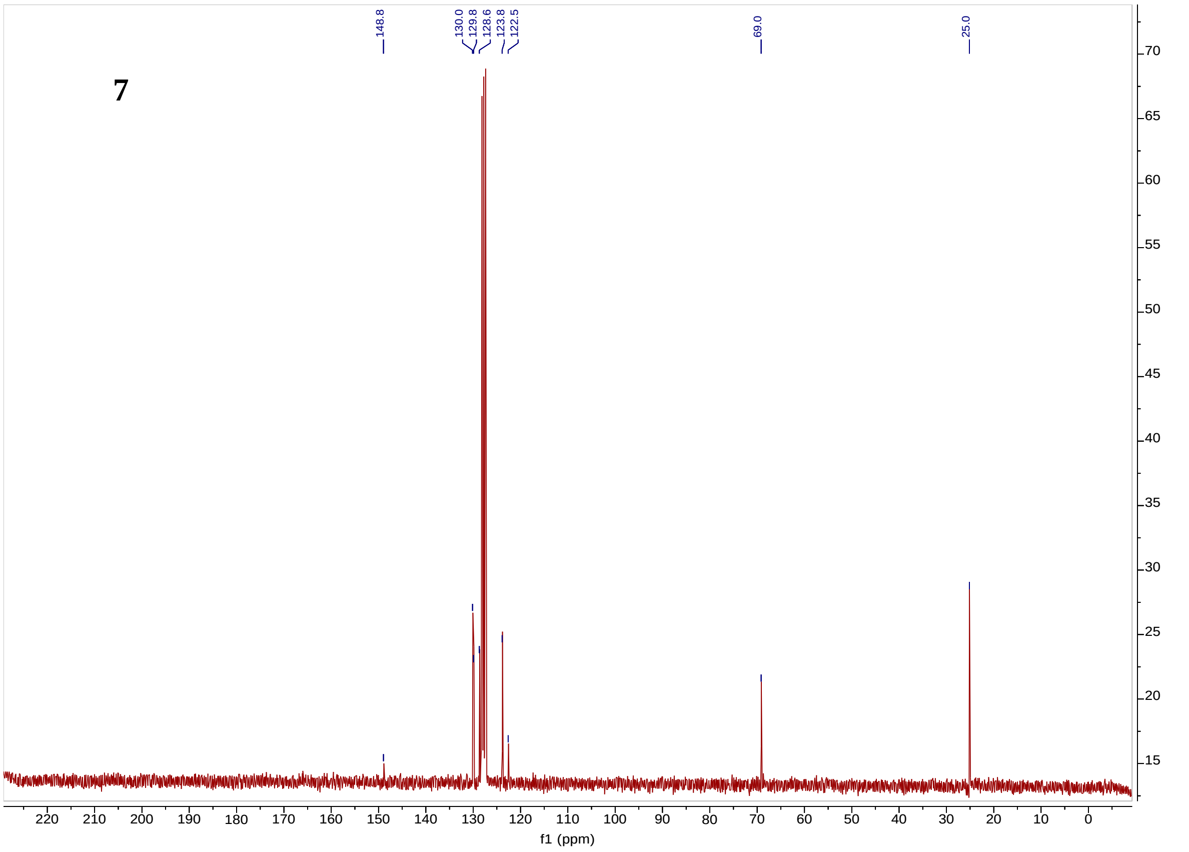
148.8
130.0
129.8
128.6
123.8
122.5
69.0
25.0
7
f1 (ppm)

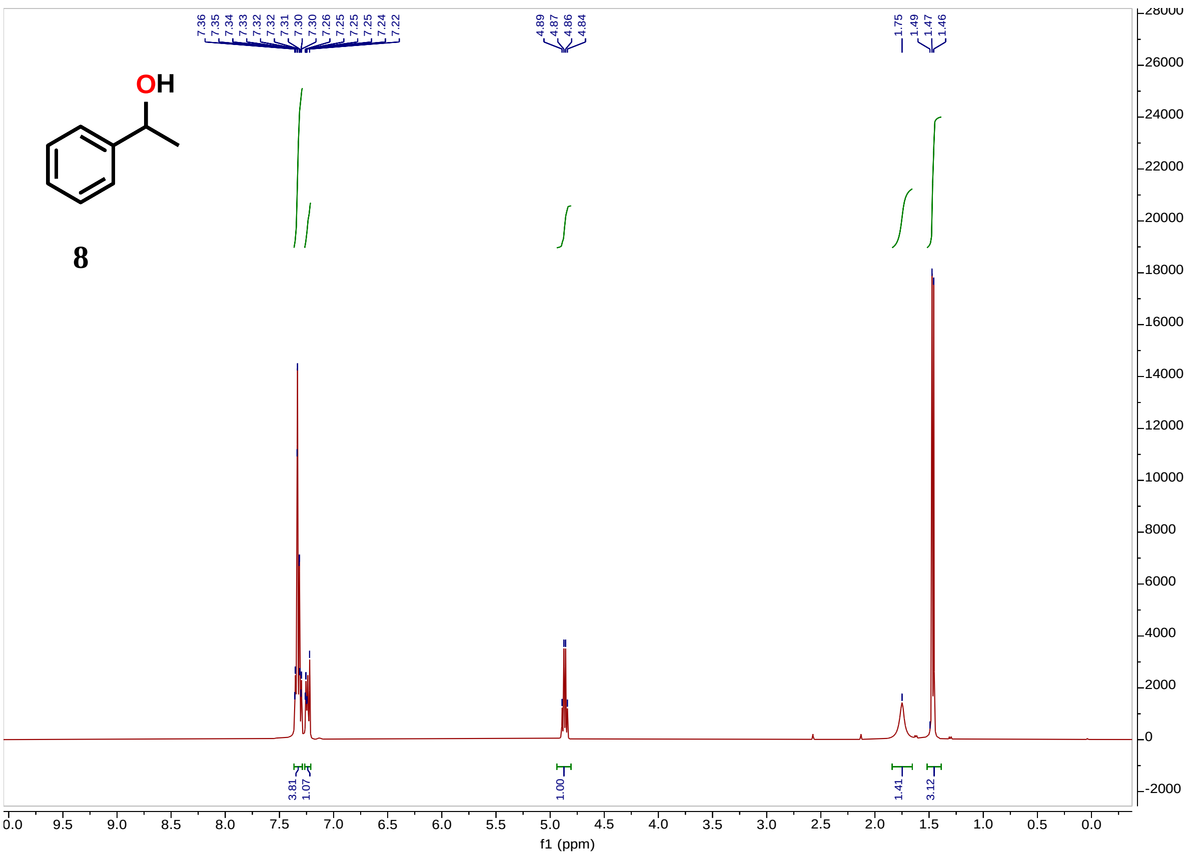

OH
8
7.36 7.35 7.34 7.33 7.32 7.32 7.31 7.30 7.30 7.26 7.25 7.25 7.25 7.24 7.22
4.89 4.87 4.86 4.84
1.75 1.49 1.47 1.46
3.81 1.07 1.00 1.41 3.12
f1 (ppm)

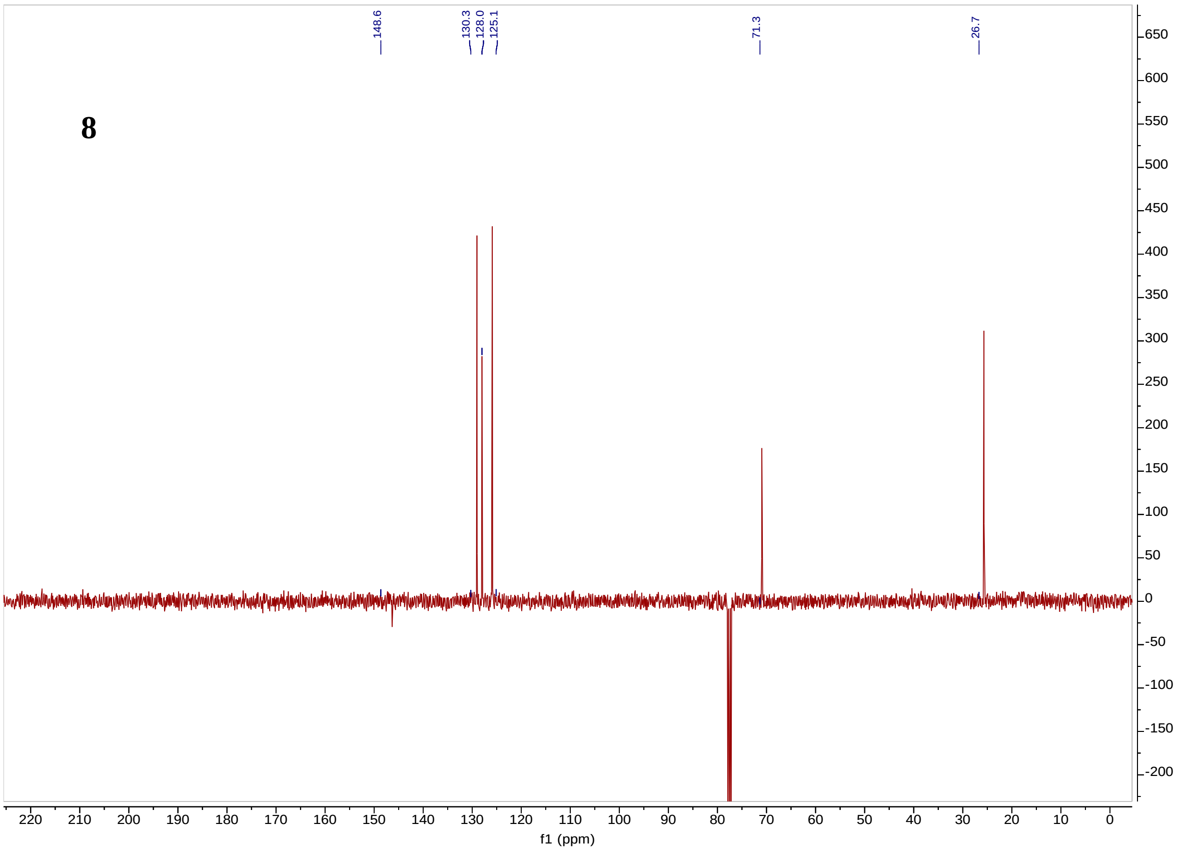

8
148.6
130.3 128.0 125.1
71.3
26.7
f1 (ppm)

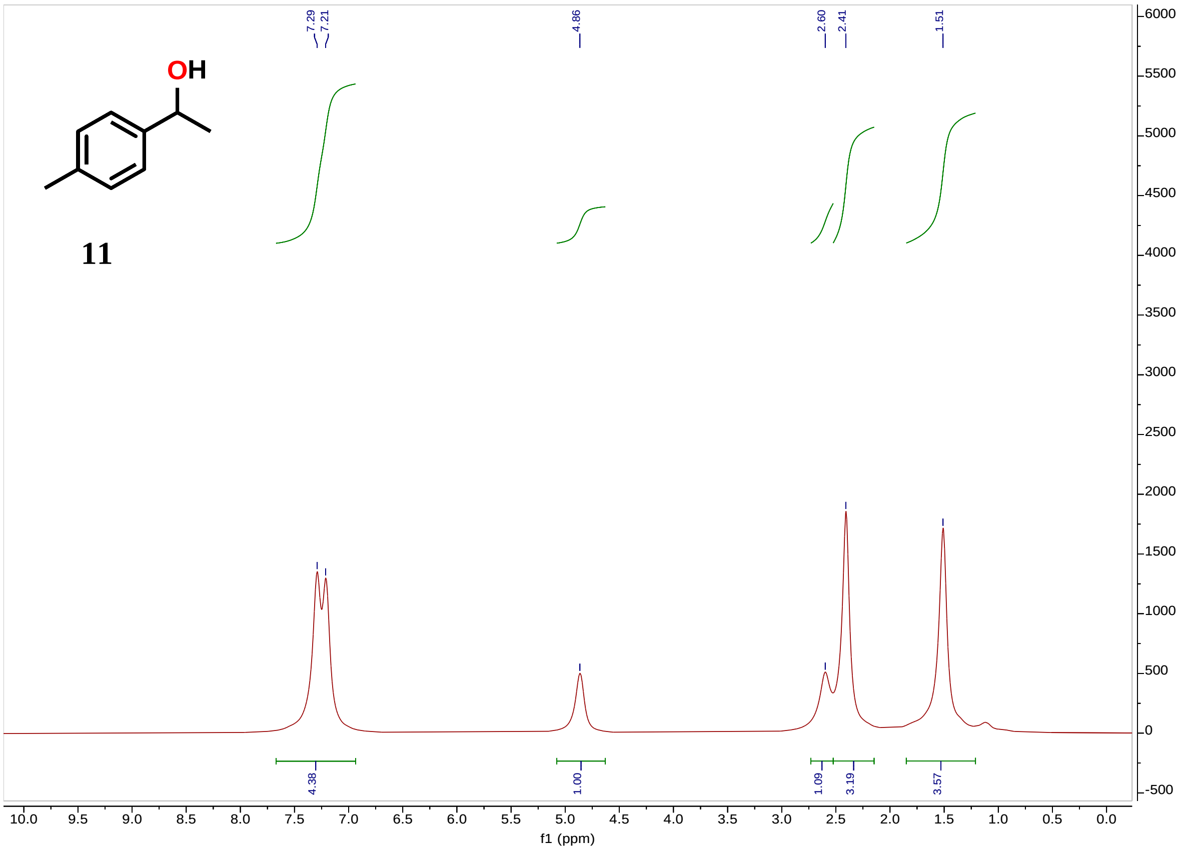

OH
11
7.29
7.21
4.86
2.60
2.41
1.51
4.38
1.00
1.09
3.19
3.57
f1 (ppm)

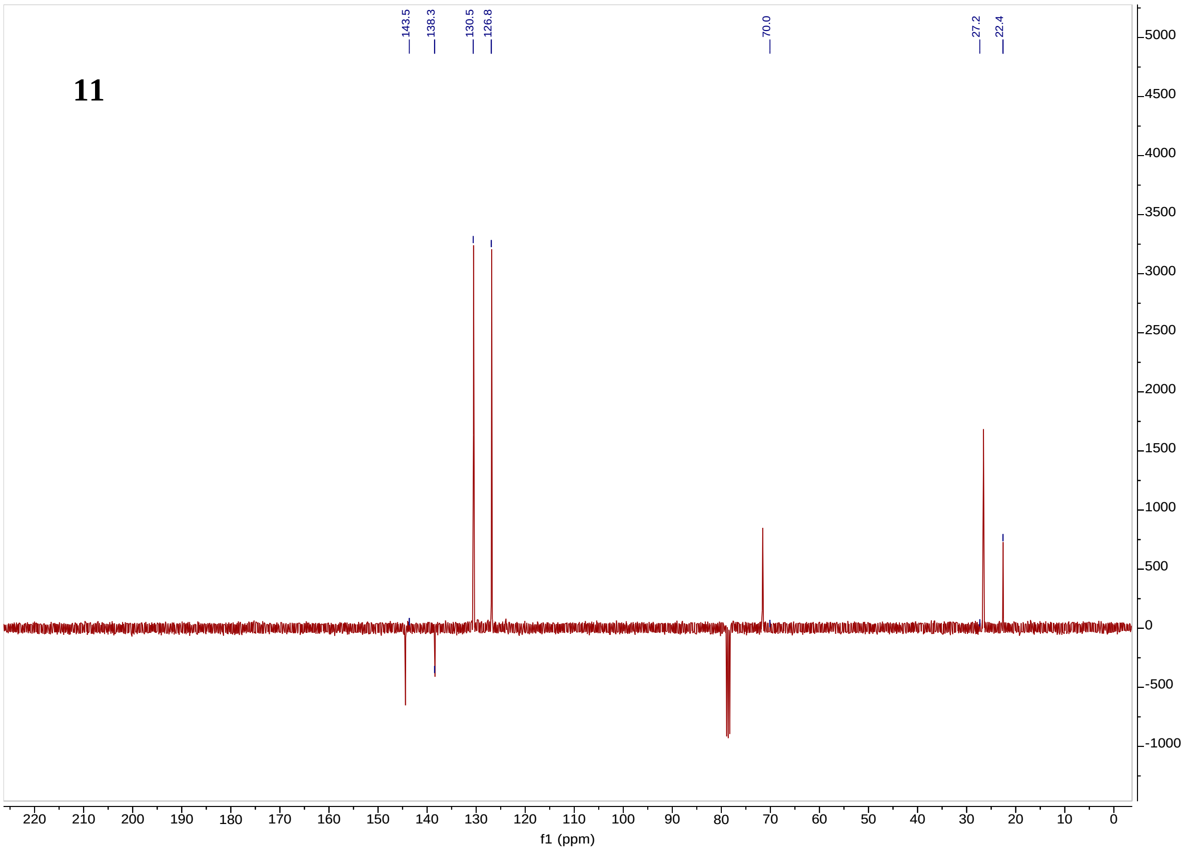

11
143.5
138.3
130.5
126.8
70.0
27.2
22.4
f1 (ppm)

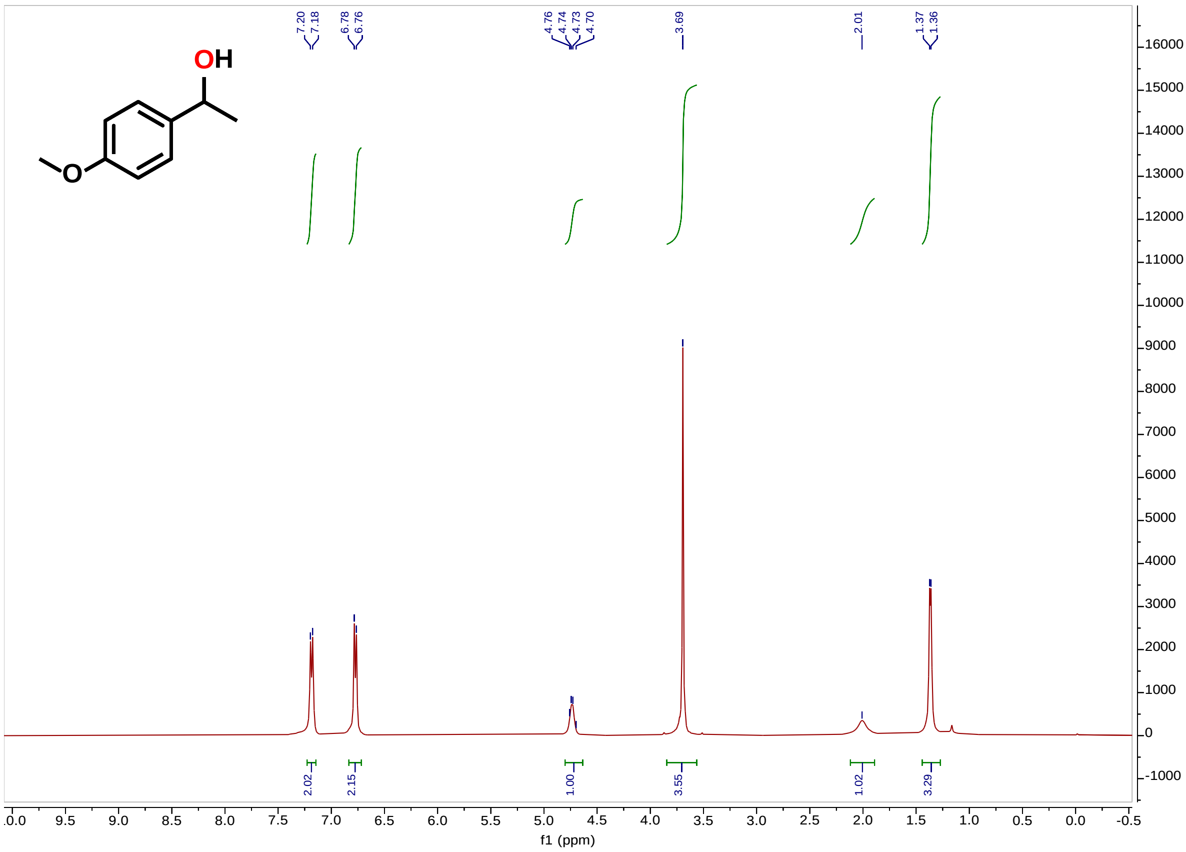
OH
O
7.20
7.18
6.78
6.76
4.76
4.74
4.73
4.70
3.69
2.01
1.37
1.36
2.02
2.15
1.00
3.55
1.02
3.29
f1 (ppm)

12

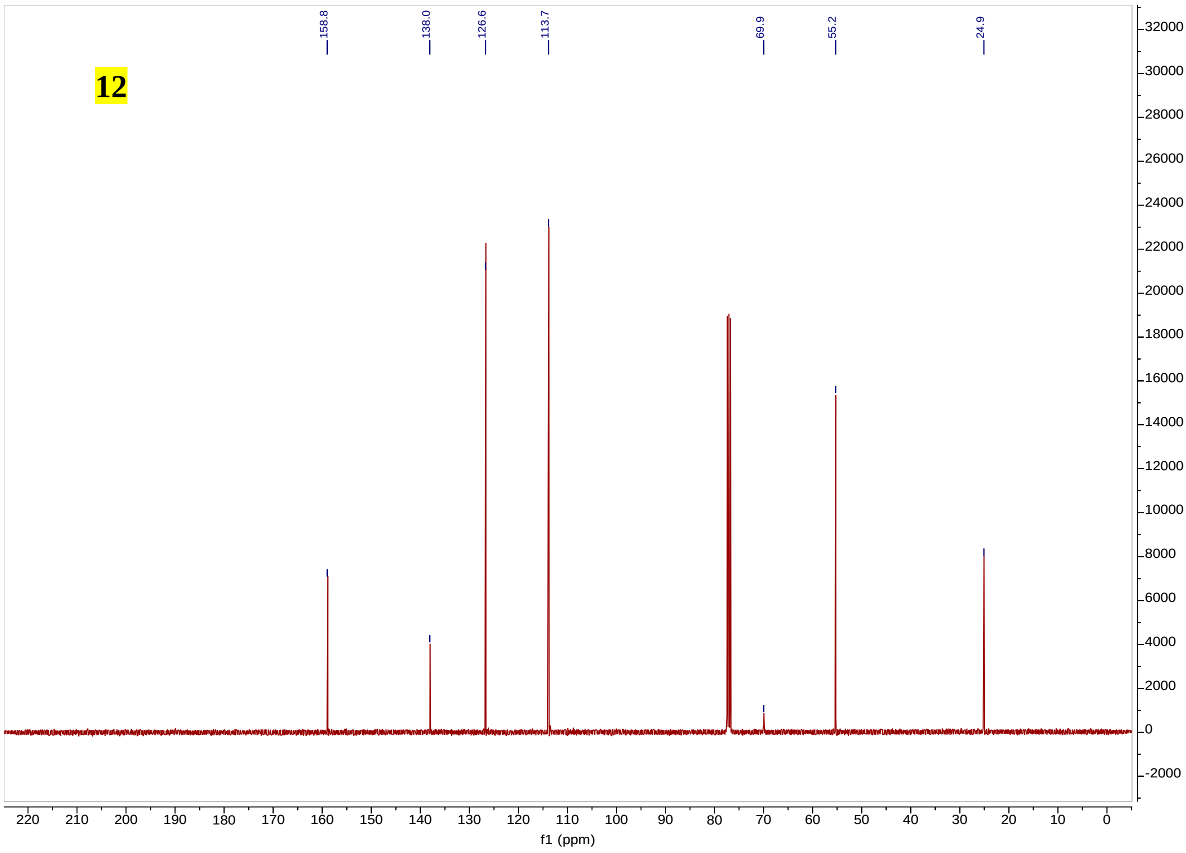
158.8
138.0
126.6
113.7
69.9
55.2
24.9
f1 (ppm)

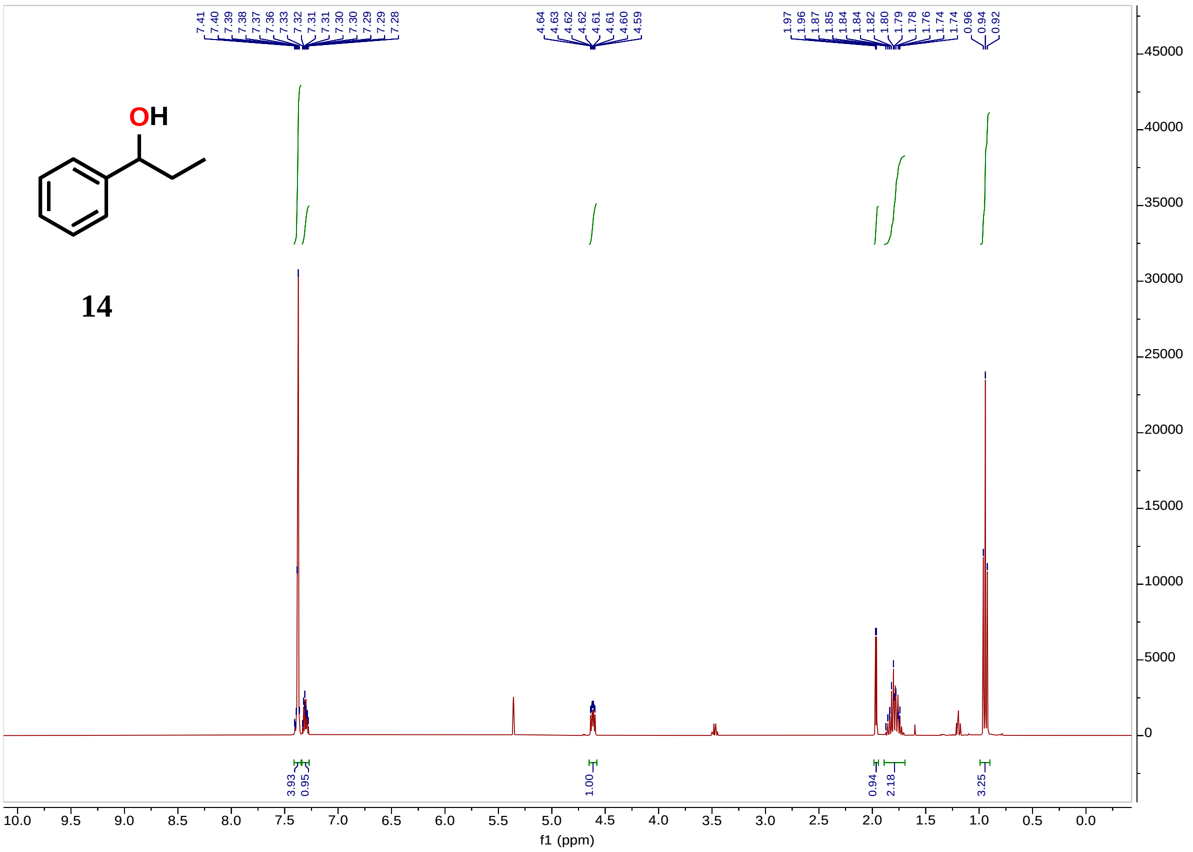
OH
14
7.41
7.40
7.39
7.38
7.37
7.36
7.33
7.32
7.31
7.31
7.30
7.30
7.29
7.29
7.28
4.64
4.63
4.62
4.62
4.61
4.61
4.60
4.59
1.97
1.96
1.87
1.85
1.84
1.84
1.82
1.80
1.79
1.78
1.76
1.74
0.96
0.94
0.92
3.93
0.95
1.00
0.94
2.18
3.25
f1 (ppm)

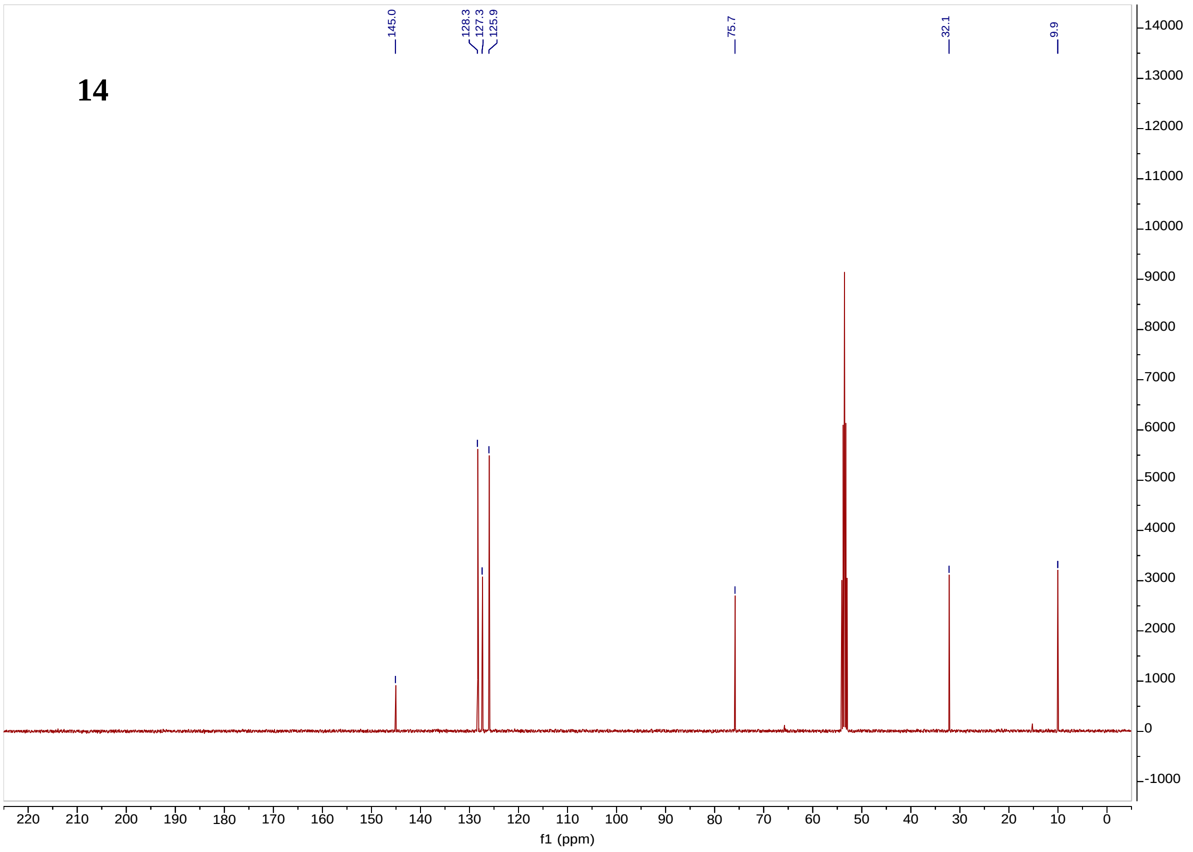
14
145.0
128.3
127.3
125.9
75.7
32.1
9.9
f1 (ppm)

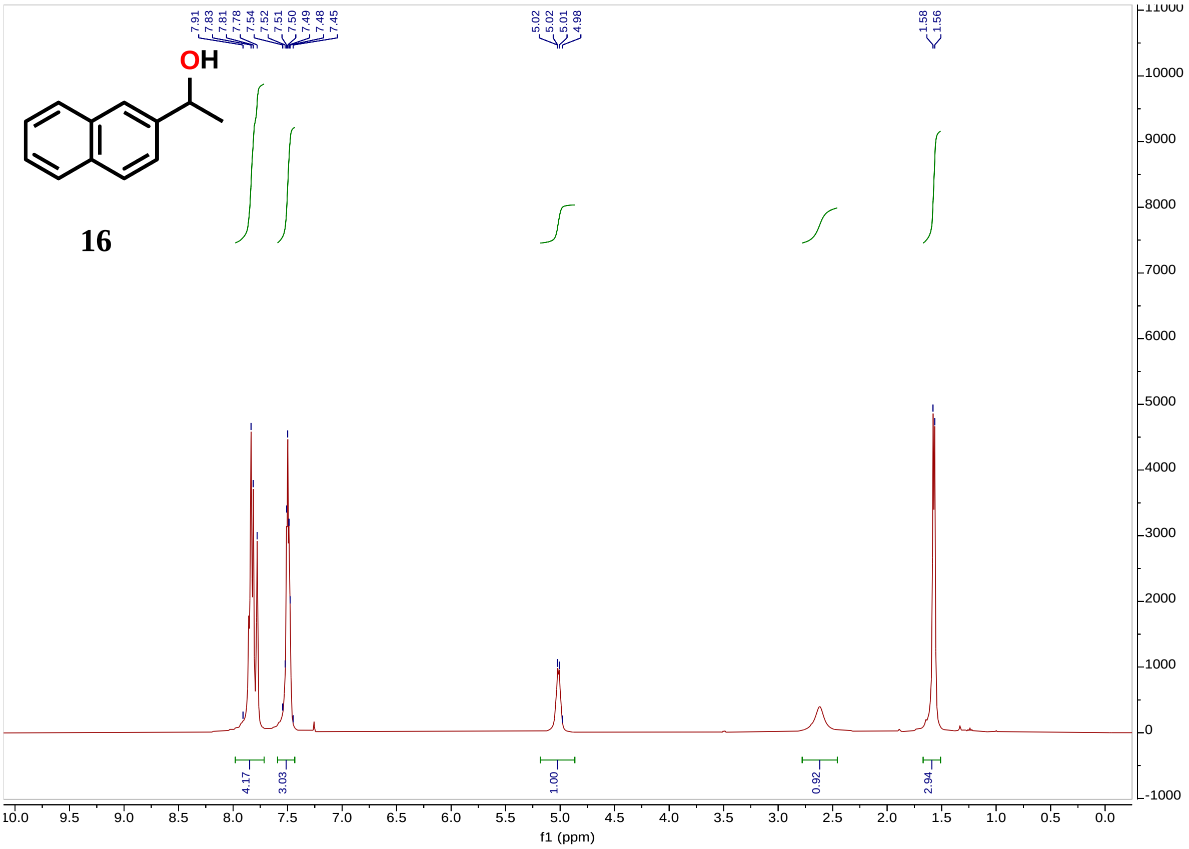

7.91 7.83 7.81 7.78 7.54 7.52 7.51 7.50 7.49 7.48 7.45
5.02 5.02 5.01 4.98
1.58 1.56
OH
16
4.17
3.03
1.00
0.92
2.94
f1 (ppm)

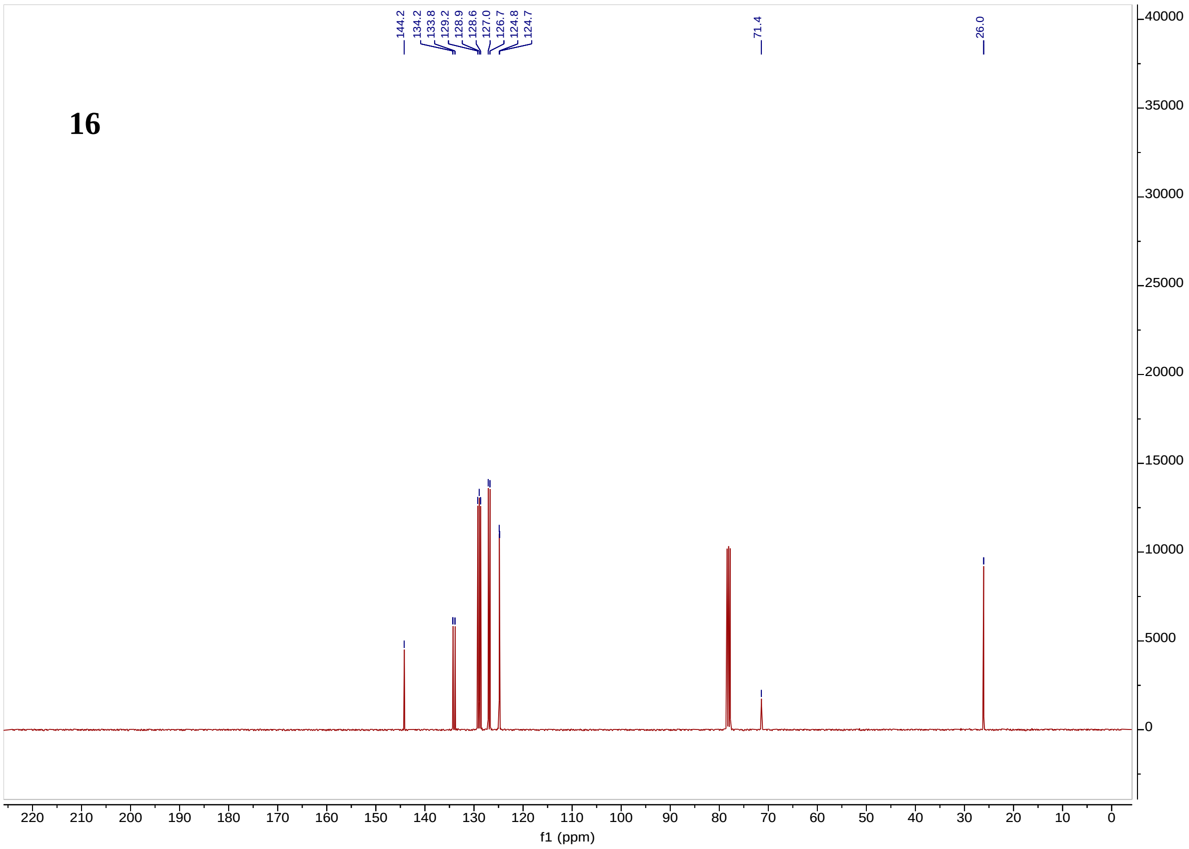

144.2 134.2 133.8 129.2 128.9 128.6 127.0 126.7 124.8 124.7
71.4
26.0
16
f1 (ppm)

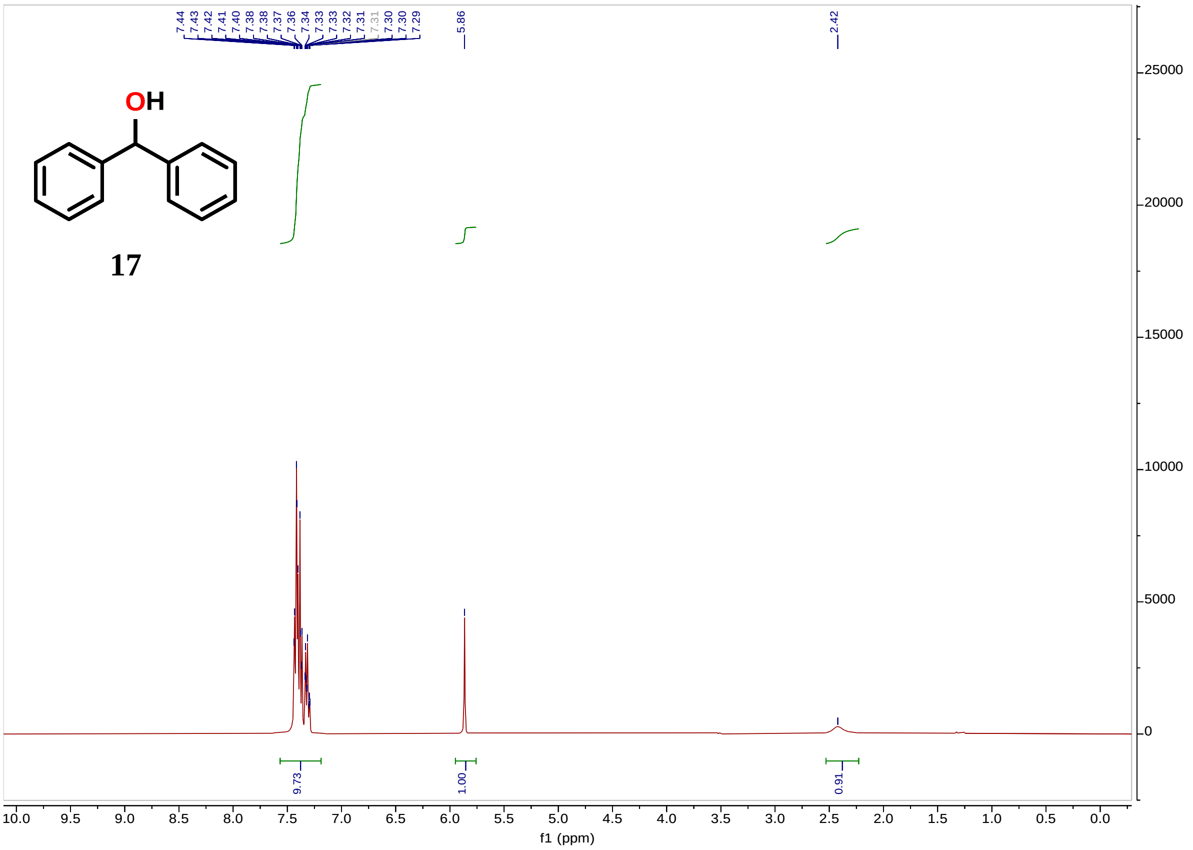
7.44 7.43 7.42 7.41 7.40 7.38 7.38 7.37 7.36 7.34 7.33 7.33 7.32 7.31 7.31 7.30 7.30 7.29
5.86
2.42
OH
17
9.73
1.00
0.91
f1 (ppm)

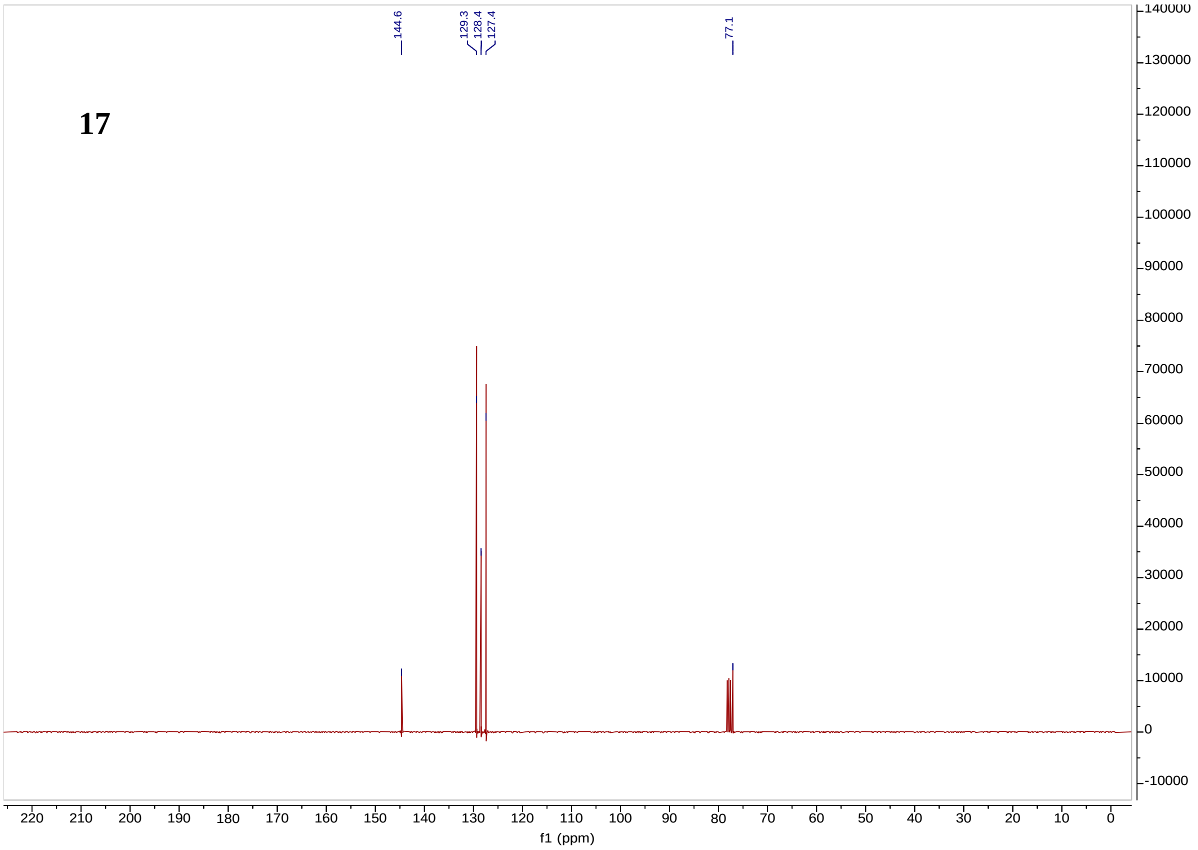
144.6
129.3
128.4
127.4
77.1
17
f1 (ppm)

18

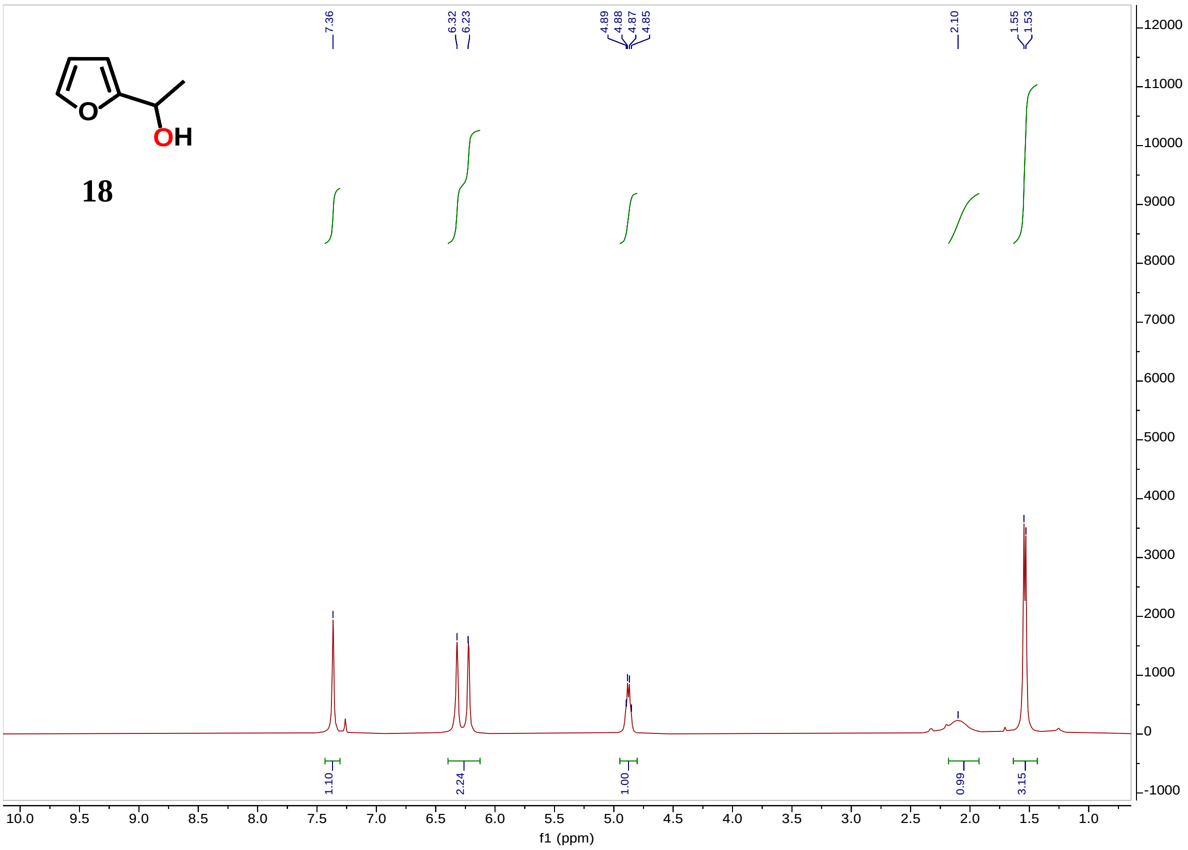

7.36
6.32
6.23
4.89
4.88
4.87
4.85
2.10
1.55
1.53
1.10
2.24
1.00
0.99
3.15
f1 (ppm)

18

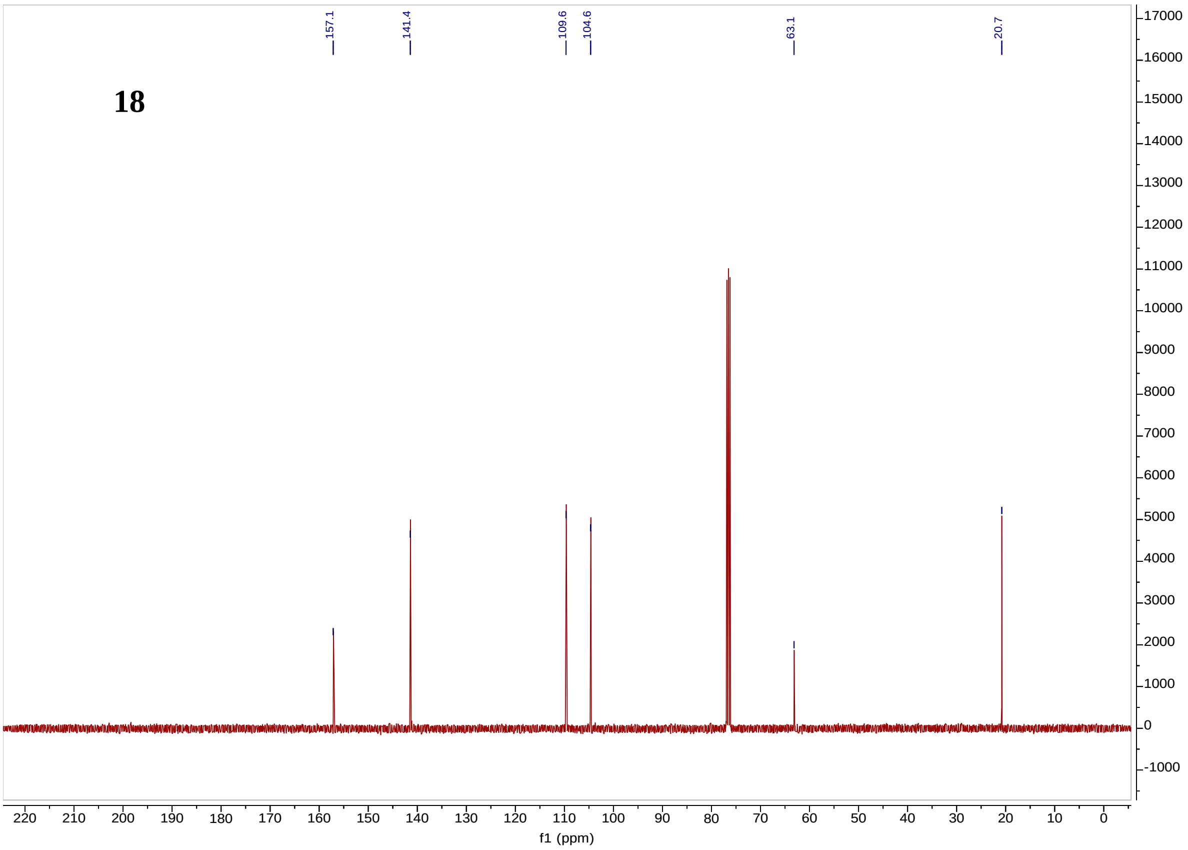

157.1
141.4
109.6
104.6
63.1
20.7
f1 (ppm)

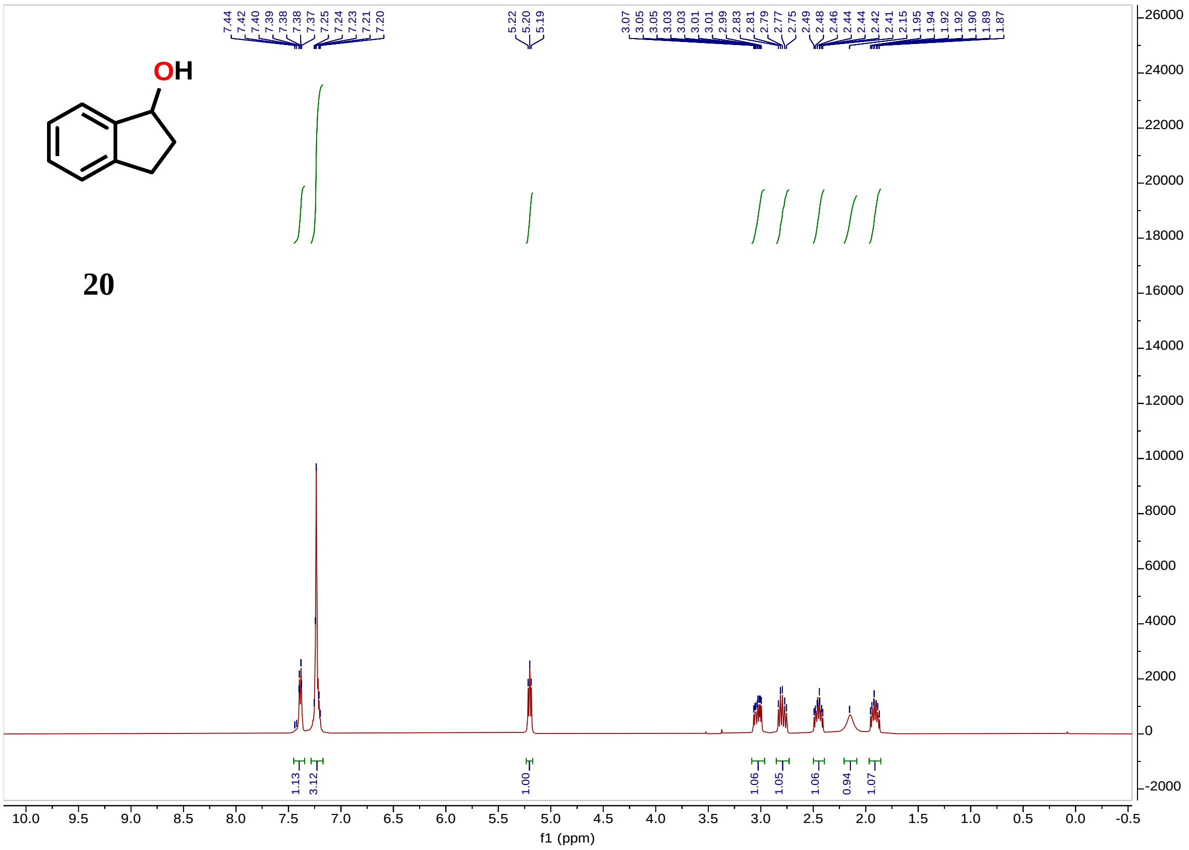
OH
20
f1 (ppm)

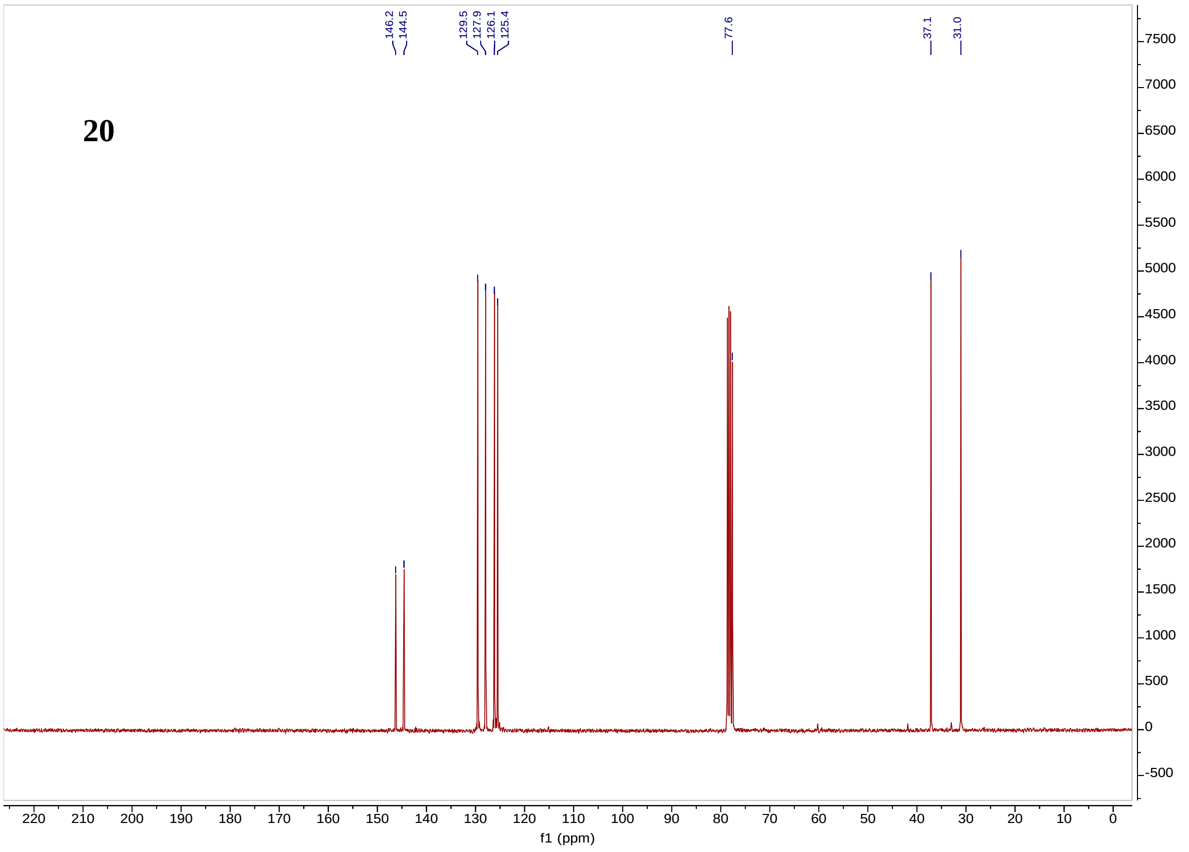
20
f1 (ppm)

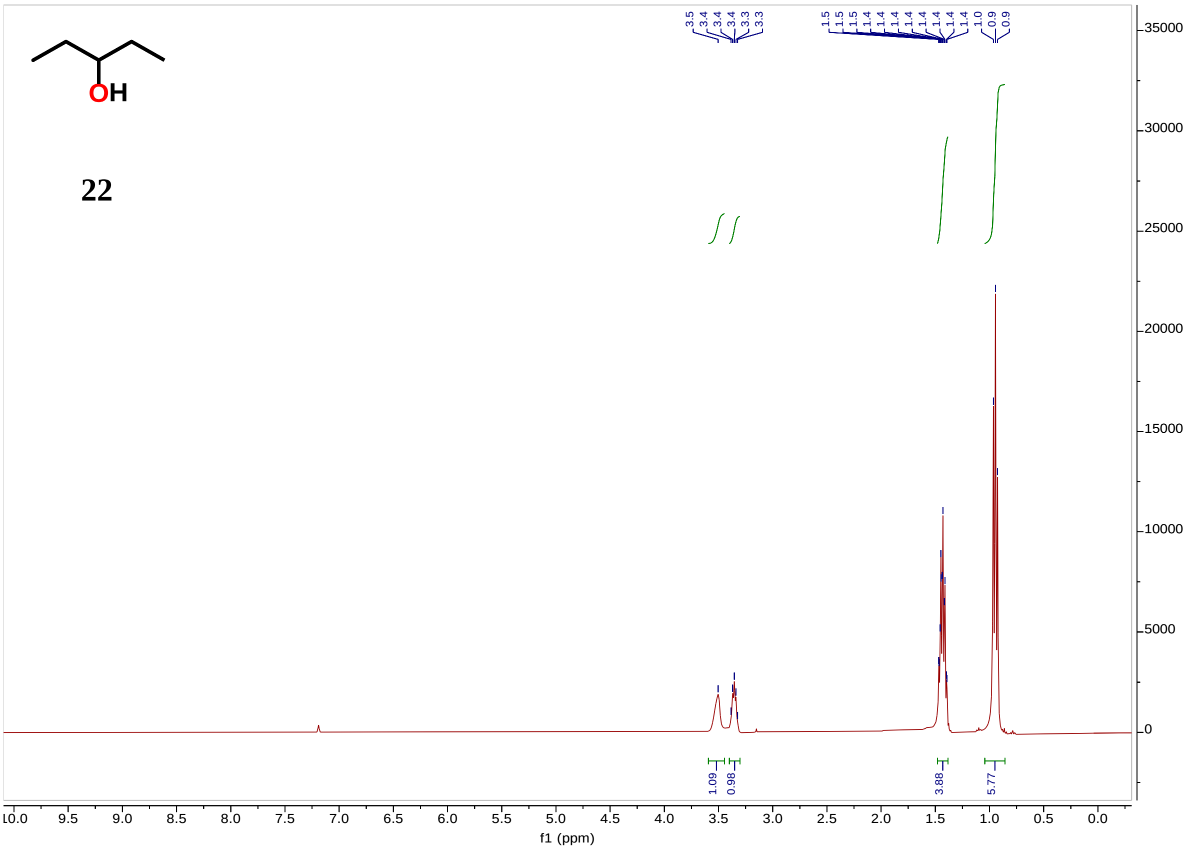
22
1.09
0.98
3.88
5.77
f1 (ppm)

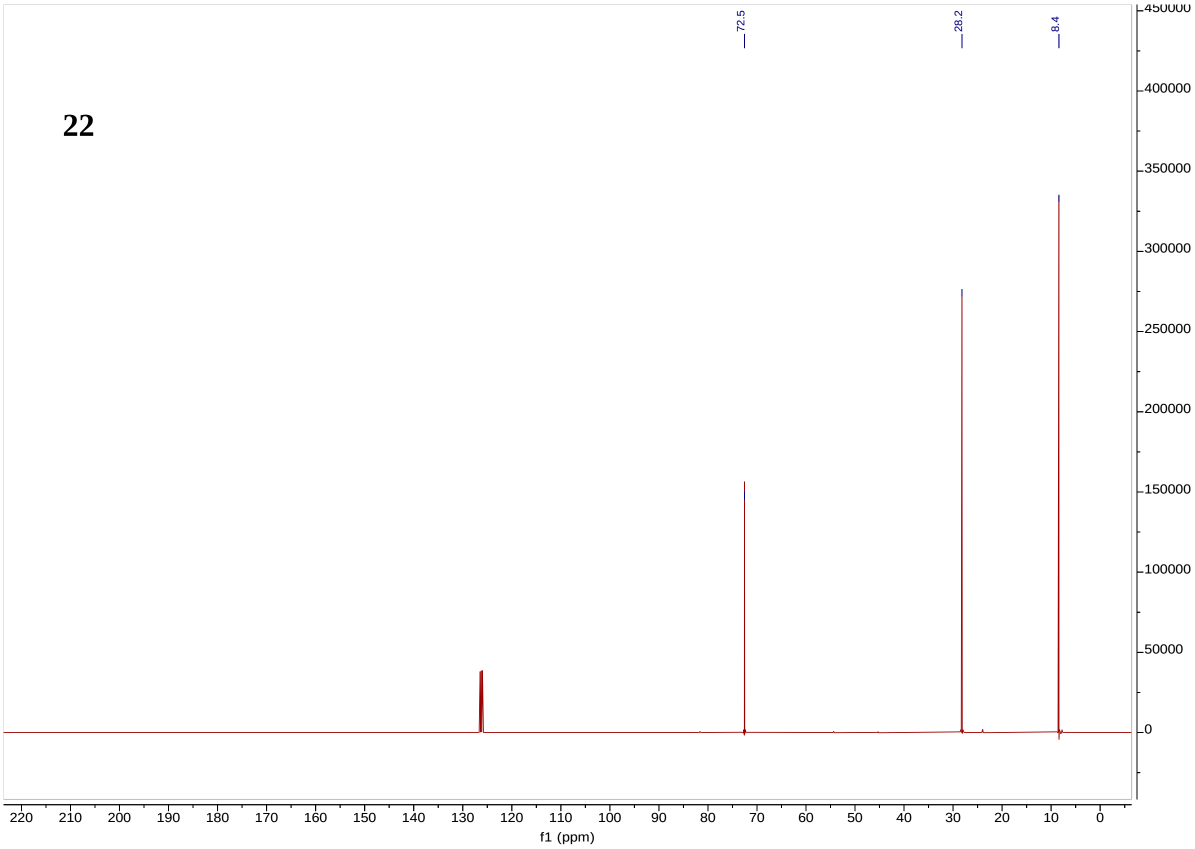
22
72.5
28.2
8.4
f1 (ppm)

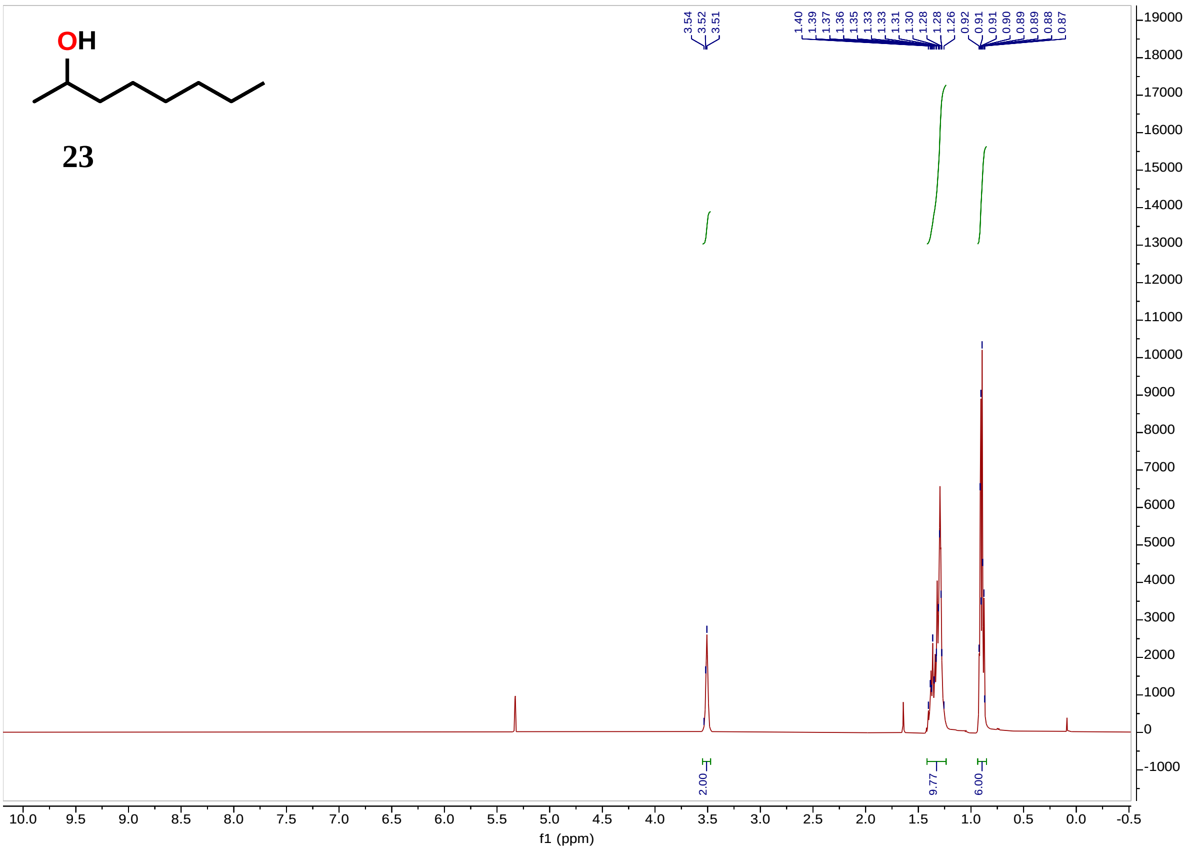
OH
23
3.54
3.52
3.51
1.40
1.39
1.37
1.36
1.35
1.33
1.33
1.31
1.30
1.28
1.28
1.26
0.92
0.91
0.91
0.90
0.89
0.89
0.88
0.87
2.00
9.77
6.00
f1 (ppm)

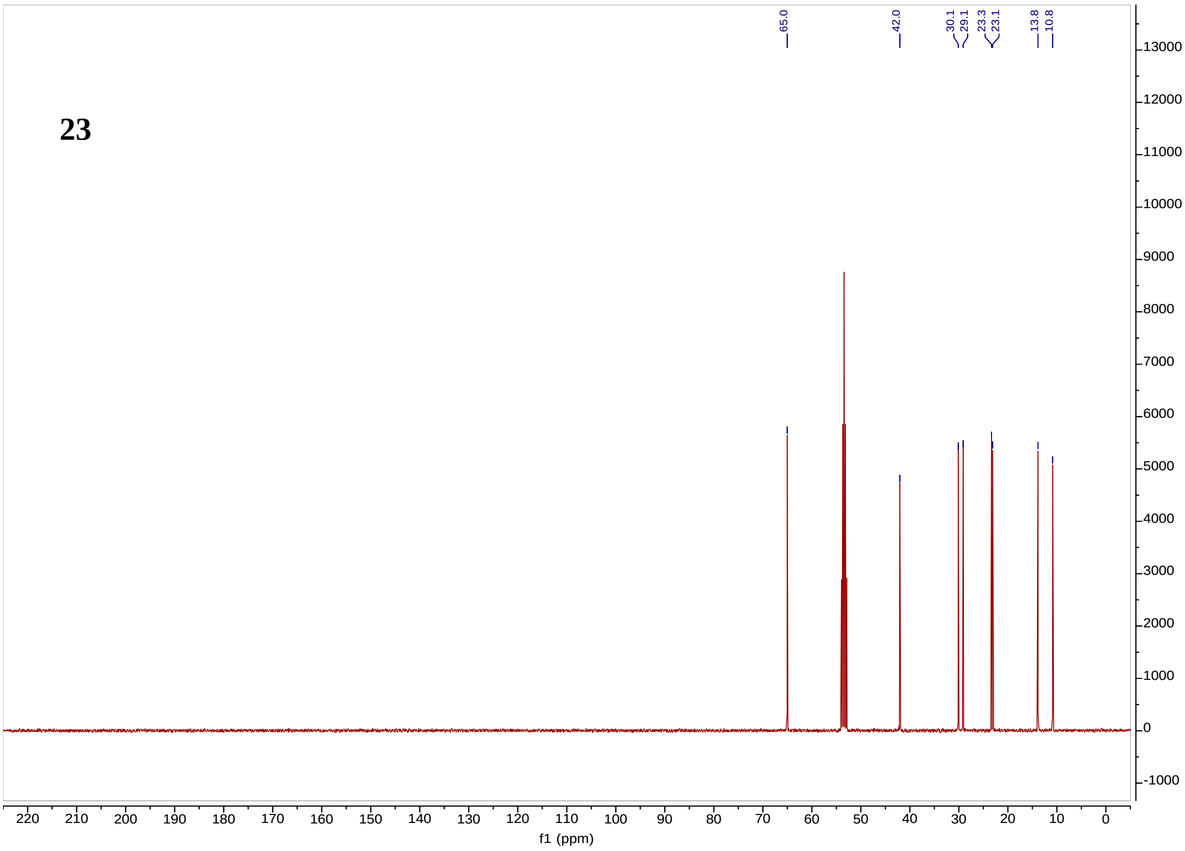
23
65.0
42.0
30.1
29.1
23.3
23.1
13.8
10.8
f1 (ppm)

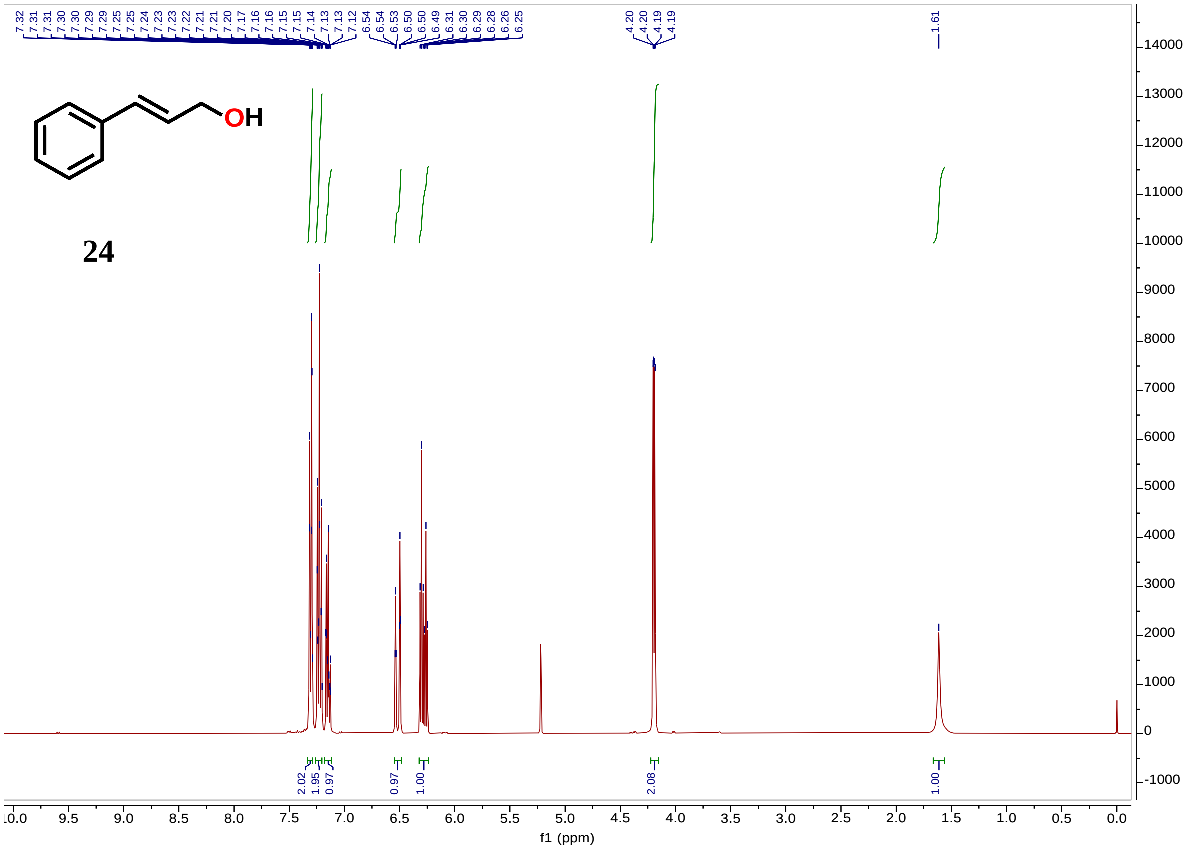

7.32 7.31 7.31 7.30 7.30 7.29 7.29 7.25 7.25 7.24 7.23 7.23 7.22 7.21 7.21 7.20 7.17 7.16 7.16 7.15 7.15 7.14 7.13 7.13 7.12 6.54 6.54 6.53 6.50 6.50 6.49 6.31 6.30 6.30 6.29 6.28 6.26 6.25
4.20 4.20 4.19 4.19
1.61
OH
24
2.02 1.95 0.97 0.97 1.00 2.08 1.00
f1 (ppm)

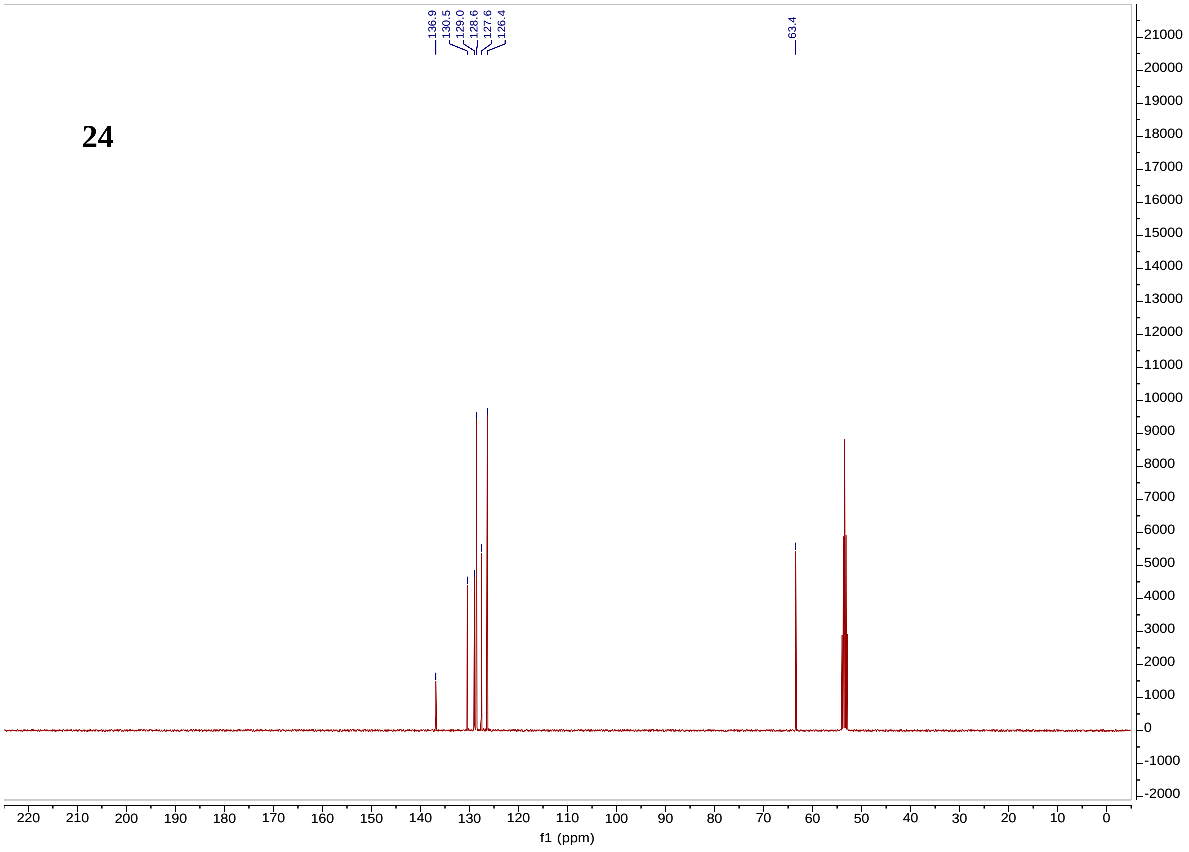

136.9 130.5 129.0 128.6 127.6 126.4
63.4
24
f1 (ppm)

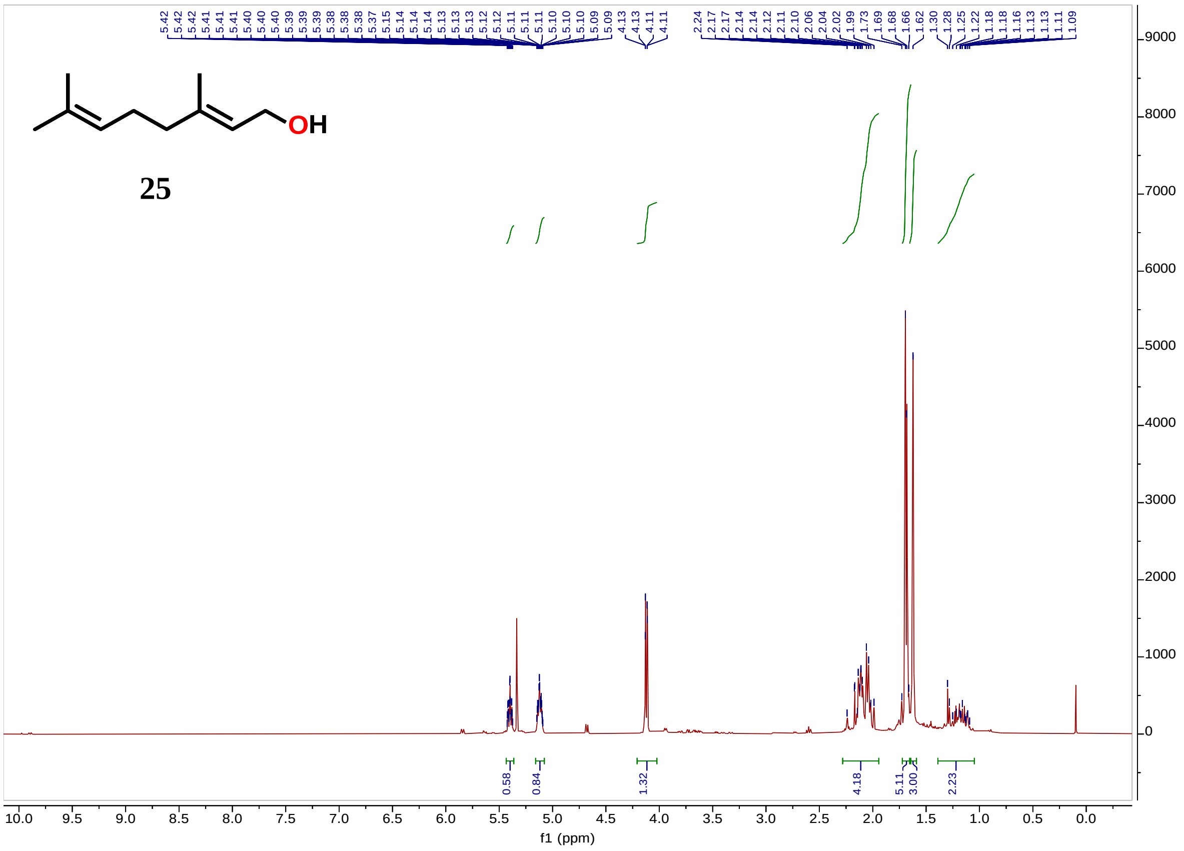
OH
25
f1 (ppm)

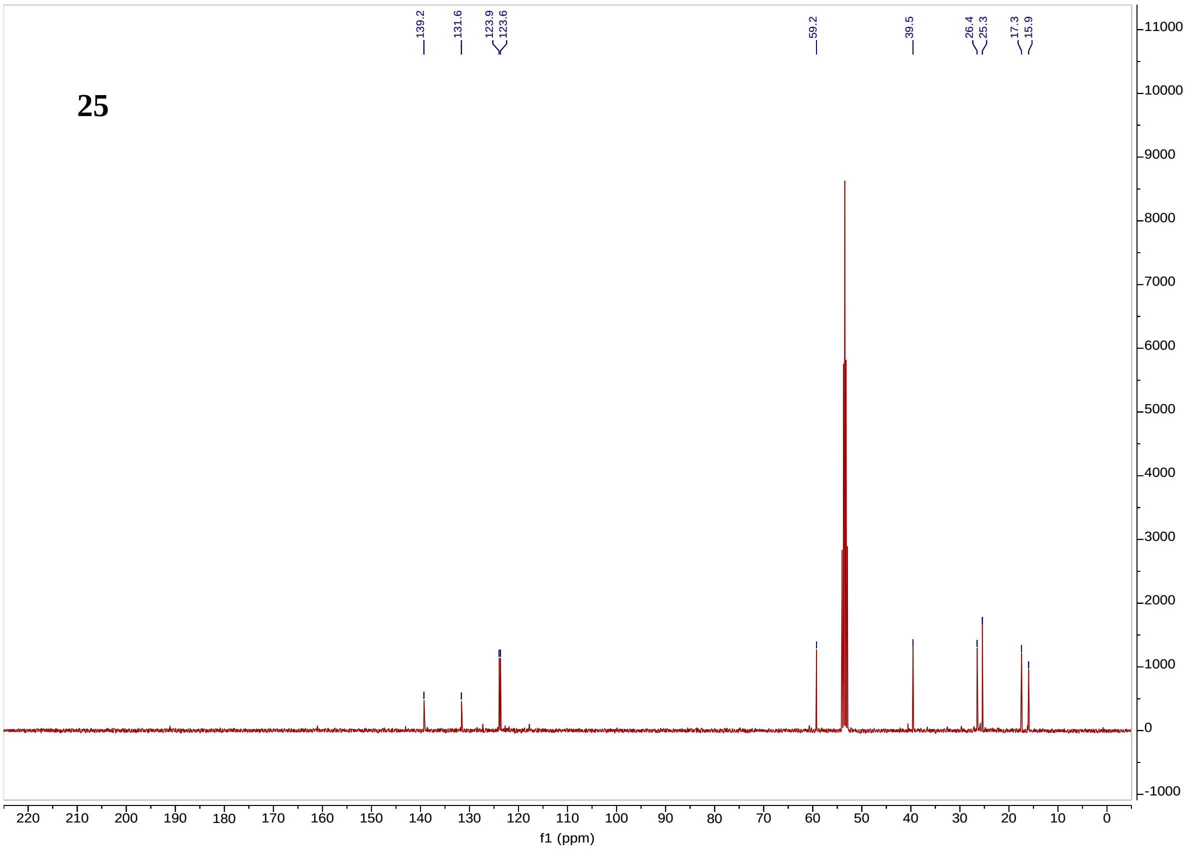
25
f1 (ppm)

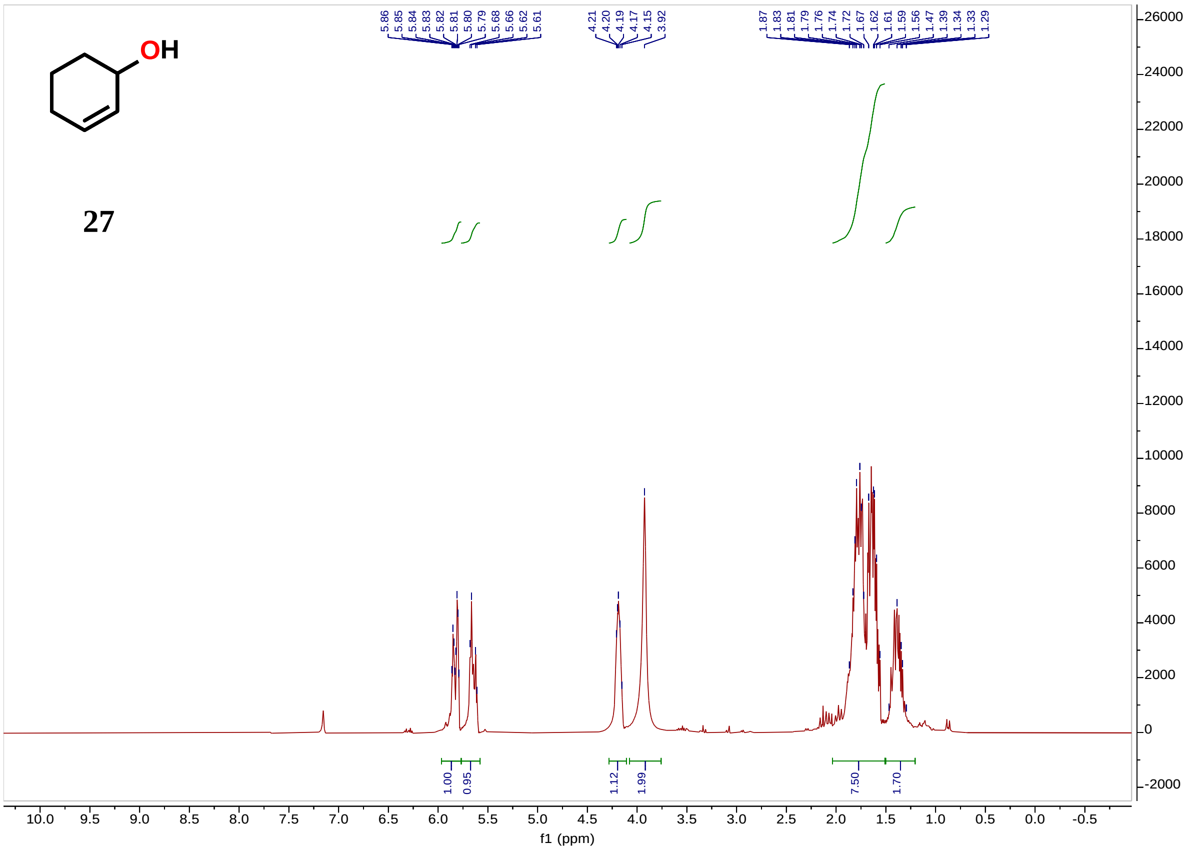
OH
27
5.86 5.85 5.84 5.83 5.82 5.81 5.80 5.79 5.68 5.66 5.62 5.61
4.21 4.20 4.19 4.17 4.15 3.92
1.87 1.83 1.81 1.79 1.76 1.74 1.72 1.67 1.62 1.61 1.59 1.56 1.47 1.39 1.34 1.33 1.29
1.00 0.95 1.12 1.99 7.50 1.70
f1 (ppm)

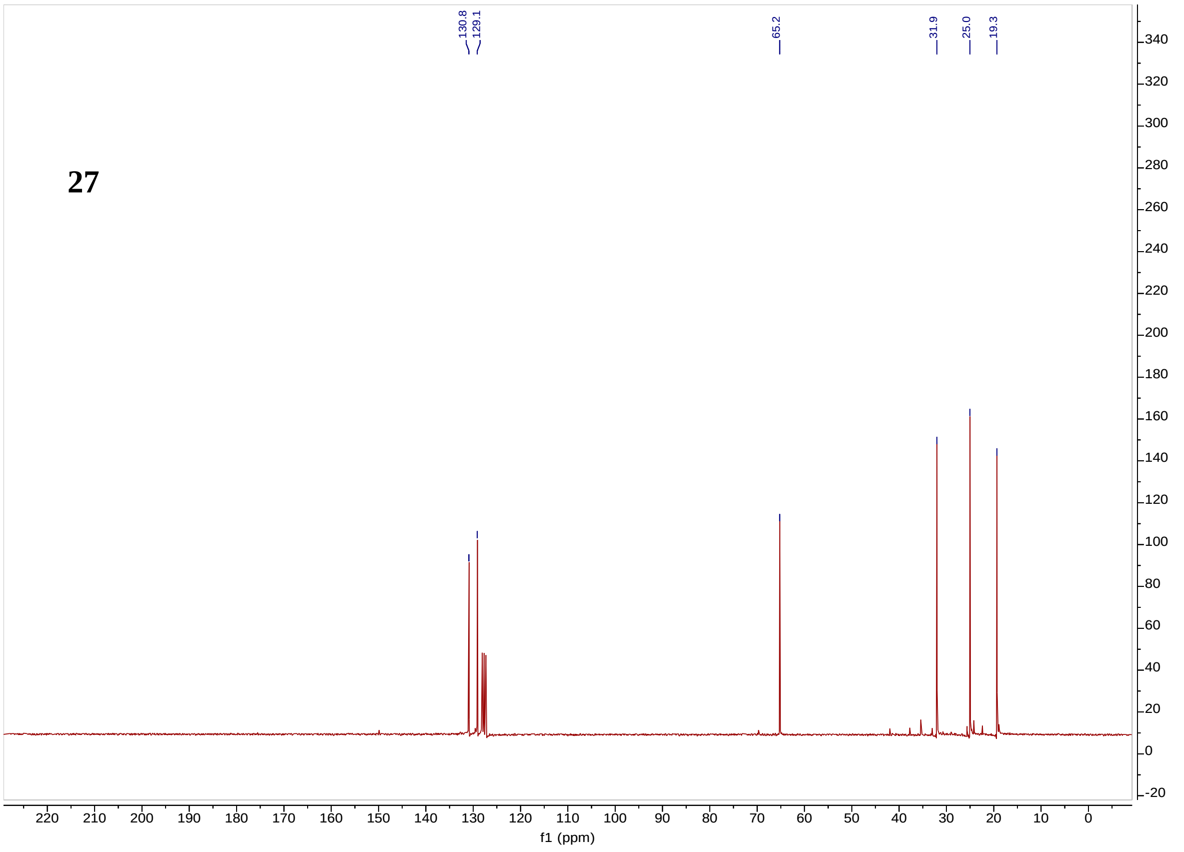
130.8 129.1
65.2
31.9 25.0 19.3
27
f1 (ppm)

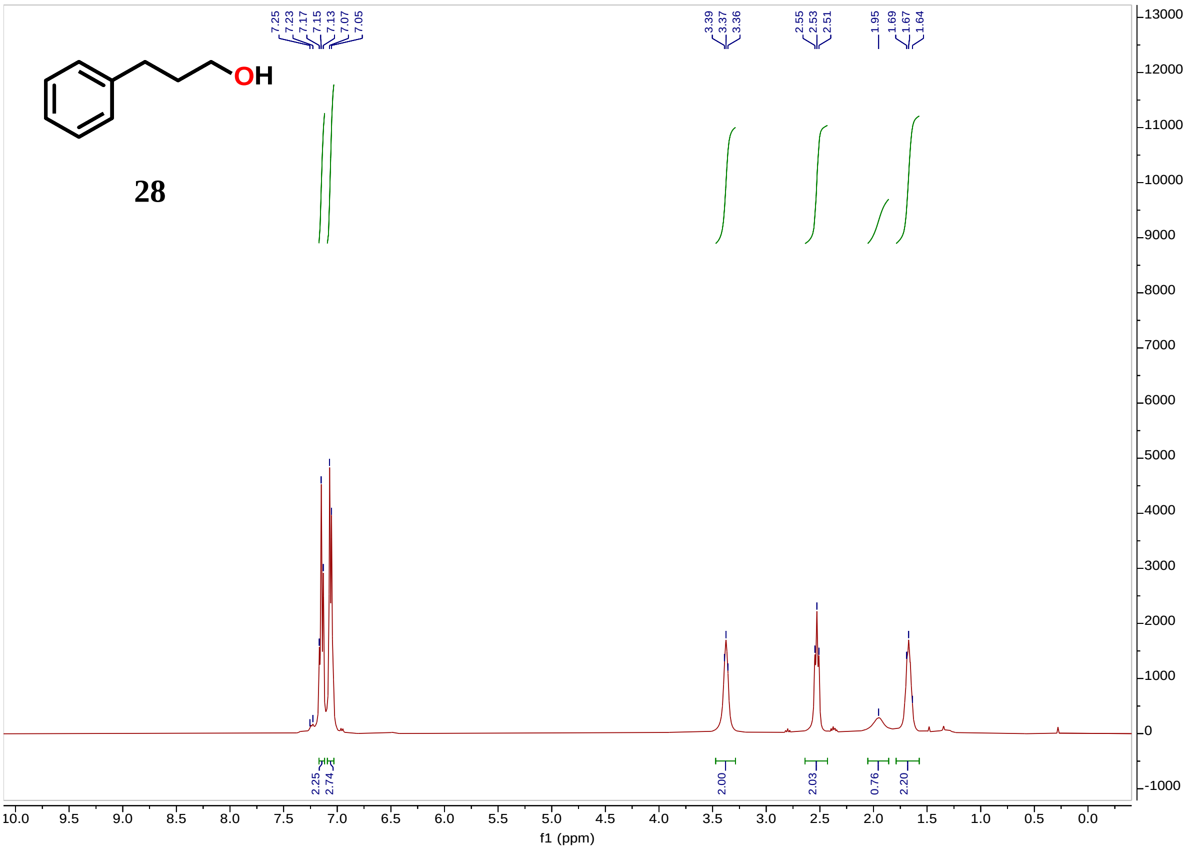
OH
28
f1 (ppm)

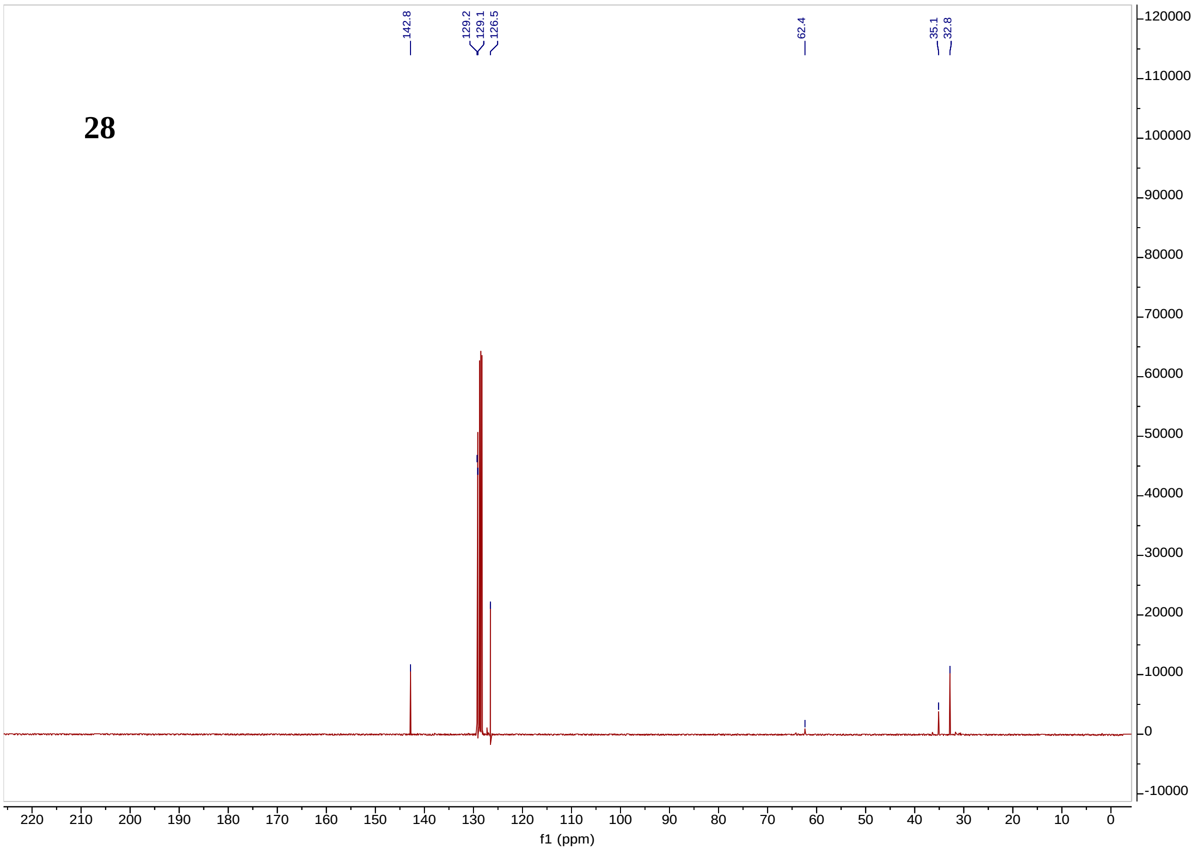
28
f1 (ppm)

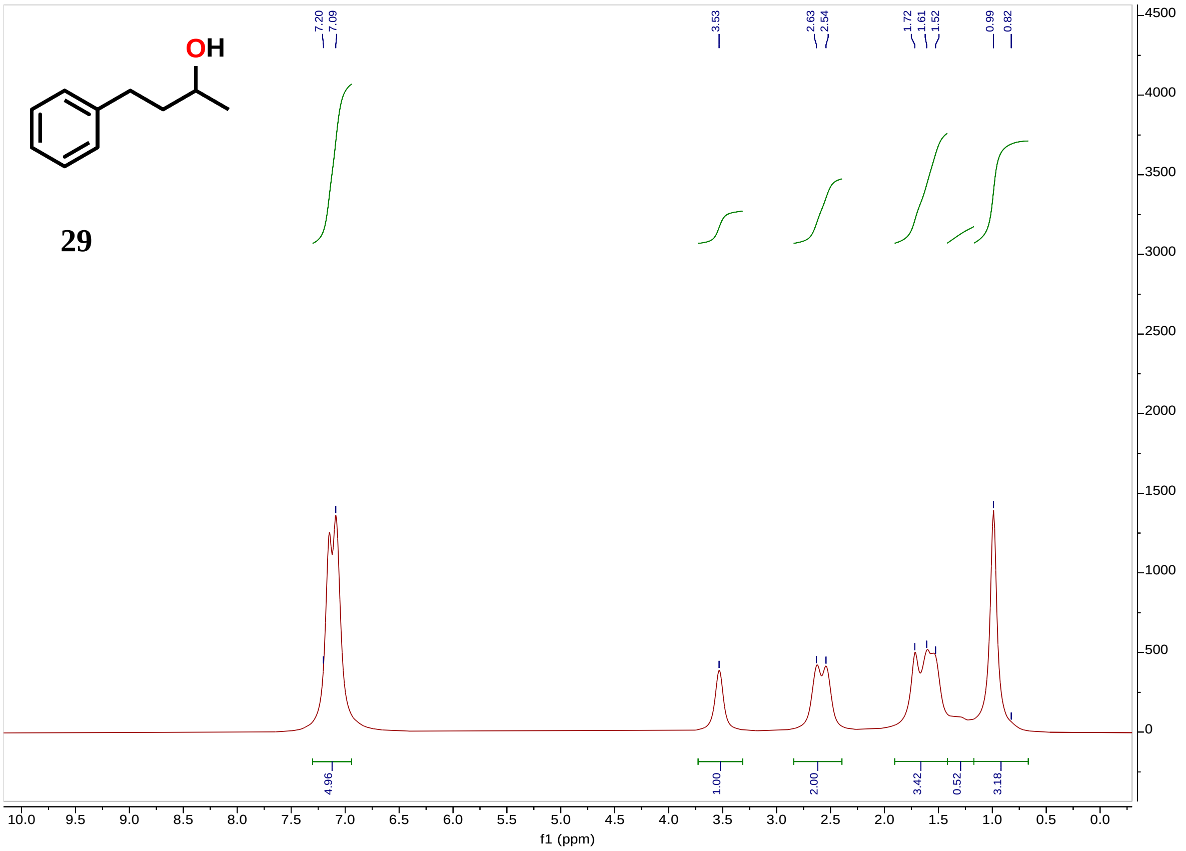
OH
29
7.20
7.09
3.53
2.63
2.54
1.72
1.61
1.52
0.99
0.82
4.96
1.00
2.00
3.42
0.52
3.18
f1 (ppm)

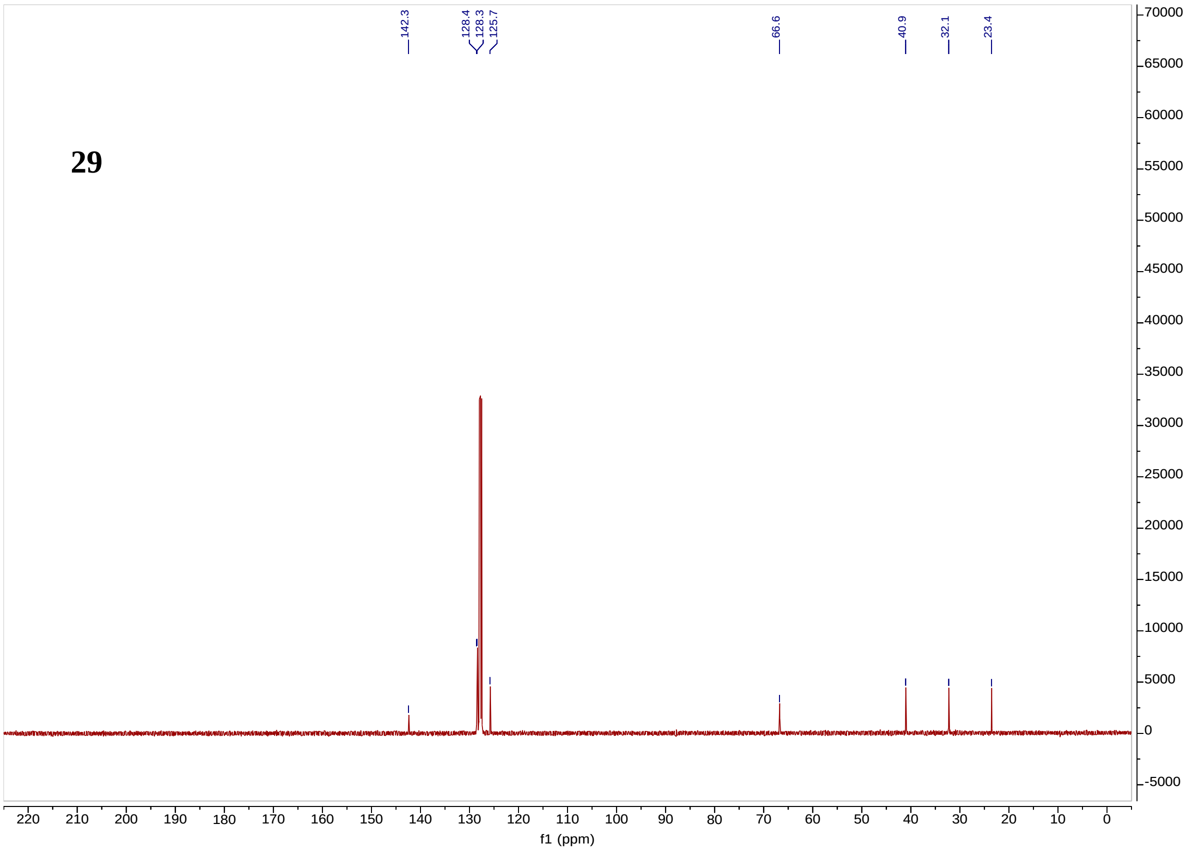
29
142.3
128.4
128.3
125.7
66.6
40.9
32.1
23.4
f1 (ppm)

3. References

(1) Perez, M.; Elangovan, S.; Spanneberg, A.; Junge, K.; Beller, M. Molecularly Defined Manganese Pincer Complexes for Selective Transfer Hydrogenation of Ketones. *ChemSusChem* **2016**, *10*, 83-86.

(2) Bruneau-Voisine, A.; Wang, D.; Dorcet, V.; Roisnel, T.; Darcel, C.; Sortais, J.-B. Transfer Hydrogenation of Carbonyl Derivatives Catalyzed by an Inexpensive Phosphine-Free Manganese Precatalyst. *Org. Lett.* **2017**, *19*, 3656-3659.

(3) Azerraf, C.; Gelman, D. Exploring the Reactivity of C(sp^3)-Cyclometalated Ir^{III} Compounds in Hydrogen Transfer Reactions. *Chem. Eur. J.* **2008**, *14*, 10364-10368.

(4) Kim, J.W., Koike, T.; Kotani, M.; Yamaguchi, K.; Mizuno, N. Synthetic Scope of $Ru(OH)_x/Al_2O_3$-Catalyzed Hydrogen-Transfer Reactions: An Application to Reduction of Allylic Alcohols by a Sequential Process of Isomerization/Meerwein–Ponndorf–Verley-Type Reduction. *Chem. Eur. J.* **2008**, *14*, 4104-4109.

(5) Weber, S.; Brünig, J.; Zeindlhofer, V.; Schröder, C.; Stöger, B.; Limbeck, A.; Kirchner, K.; Bica, K. Selective Hydrogenation of Aldehydes Using a Well-Defined Fe(II) PNP Pincer Complex in Biphasic Medium. *ChemCatChem* **2018**, *10*, 4386-4394.

(6) Trost, B.M.; Xu, J.; Reichle, M. Enantioselective Synthesis of α-Tertiary Hydroxyaldehydes by Palladium-Catalyzed Asymmetric Allylic Alkylation of Enolates. *J. Am. Chem. Soc.* **2007**, *129*, 282-283.

(7) Weber, S.; Stöger, B.; Veiros, L. F.; Kirchner, K. Rethinking Basic Concepts - Hydrogenation of Alkenes Catalyzed by Bench-Stable Alkyl Mn(I) Complexes. *ACS Catal.* 2019, *9*, 9715-9720.
